# Differential Expression and Bioinformatics Analysis of Plasma-Derived Exosomal circRNA in Type 1 Diabetes Mellitus

**DOI:** 10.1155/2022/3625052

**Published:** 2022-10-27

**Authors:** Haipeng Pang, Wenqi Fan, Xiajie Shi, Shuoming Luo, Yimeng Wang, Jian Lin, Yang Xiao, Xia Li, Gan Huang, Zhiguo Xie, Zhiguang Zhou

**Affiliations:** National Clinical Research Center for Metabolic Diseases, Key Laboratory of Diabetes Immunology (Central South University), Ministry of Education, and Department of Metabolism and Endocrinology, The Second Xiangya Hospital of Central South University, Changsha, 410011 Hunan, China

## Abstract

**Backgrounds:**

Both exosome and circular RNA (circRNA) have been reported to participate in the pathogenesis of type 1 diabetes mellitus (T1DM). However, the exact role of exosomal circRNA in T1DM is largely unknown. Here, we identified the exosomal circRNA expression profiles in the plasma of T1DM patients and explored their potential function using bioinformatics analysis. *Material and Methods*. Exosomes were extracted by the size exclusion chromatography method from plasma of 10 T1DM patients and 10 age- and sex- matched control subjects. Illumina Novaseq6000 platform was used to detect the exosomal circRNA expression profiles. Multiple bioinformatics analysis was applied to investigate the potential biological functions of exosomal circRNAs.

**Results:**

A total of 784 differentially expressed exosomal circRNAs have been identified in T1DM patients, of which 528 were upregulated and 256 were downregulated. Gene Ontology analysis enriched terms such as protein ubiquitination involved in ubiquitin-dependent protein catabolic protein (GO:0042787), membrane (GO:0016020), and GTPase activator activity (GO:0005096). The most enriched pathway in Kyoto Encyclopedia of Genes and Genomes was ubiquitin-mediated proteolysis (ko04120). The miRNA-targeting prediction method was used to identify the miRNAs that bind to circRNAs, and circRNA-miRNA-mRNA pathways were constructed, indicating that interactions between circRNA, miRNA, and gene might be involved in the disease progression.

**Conclusions:**

The present study identified the exosomal circRNA expression profiles in T1DM for the first time. Our results threw novel insights into the molecular mechanisms of T1DM.

## 1. Introduction

Type 1 diabetes mellitus (T1DM) is a chronic disease caused by the autoimmune attack against the pancreatic beta-cells [1]. The incidence and prevalence of T1DM have been increasing worldwide, and this disease remains incurable nowadays [2, 3]. Besides, T1DM can lead to multiple serious complications such as kidney failure, cardiovascular disease, and blindness, which impose tremendous physical and economic burdens among patients. Currently, it has reached a consensus that the disease condition of T1DM is triggered by environmental factors in individuals who have a genetic predisposition [4, 5]. However, the exact pathogenic mechanisms of T1DM have not been elucidated.

In recent years, mounting studies have indicated exosome and circular RNA (circRNA) are involved in the onset and development of T1DM [6, 7]. Exosomes are small (30-200 nm in diameter) extracellular vesicles (EVs) that can be released by virtually all cell types. It has been demonstrated that exosome might play an important role in mediating intercellular or interorgan crosstalk via its content such as RNA [8, 9]. Besides, exosomes are present in various kinds of body fluids and the RNA content of exosomes are strictly regulated in response to different endogenous and exogenous stimulations [7]. Therefore, the exosome might be a promising biomarker for multiple disease conditions. Recent evidence has demonstrated that exosomes participated in the progression of T1DM via multiple mechanisms. It has been indicated that T lymphocyte exosomes could trigger beta-cell apoptosis via the exosomal miRNAs [10]. Besides, the pancreatic islets could release the intracellular beta-cell autoantigens in exosomes, which could be taken up by and activate antigen-presenting cells [11]. Other studies emphasized the biomarker potential of exosomes in T1DM. It has been indicated that exosomal RNAs derived from human islets were differentially expressed under the treatment of proinflammatory cytokines, highlighting the biomarker potential of exosomal RNAs [12]. Furthermore, a study indicated that circulating EV miR-21-5p was increased during T1DM development and might be a promising maker of T1DM [13]. Our previous studies characterized the lncRNA and mRNA expression profiles in T1DM and identified several exosomal mRNAs related to T1DM progression [14, 15]. Moreover, some studies reported that stem cell-derived exosomes could serve as therapeutic tools for T1DM. For instance, an animal study using rat models of T1DM indicated that menstrual blood-derived mesenchymal stem cell- (MSC-) derived exosomes could enhance beta-cell regeneration and insulin secretion [16]. Besides, it has been indicated that exosomes released by adipose tissue-derived MSCs possessed immunomodulatory effects upon T lymphocytes and could ameliorate clinical symptoms of T1DM [17].

CircRNA is an important cargo carried by exosomes. CircRNA, a newly recognized group of noncoding RNA transcript, is a unique enclosed structure characterized by covalent binding between 3′- and 5′- phosphodiester bond. Existing studies have elucidated that circRNAs could modulate gene expression by sponging certain miRNAs, regulating nuclear transcription, and competing mRNA splicing [18]. In addition, the closed loop structure determines the high biological stability of circRNA, thus making them a promising biomarker in clinics. Indeed, some studies have screened the circRNA expression profiles in T1DM and the results indicated that some circRNAs might play a critical role in the progression of T1DM [6, 18].

However, there is lack of relevant research about the effects of exosomal circRNA on T1DM. Here, we reported the exosomal circRNA expression profiles derived from plasma of T1DM for the first time and explored their potential function by using bioinformatics analysis.

## 2. Material and Methods

### 2.1. Patients and Controls

Totally, 10 patients with T1DM (3 males and 7 females) and 10 healthy subjects (5 males and 5 females) were recruited from the Second Xiangya Hospital of Central South University. The average age of T1DM patients was 25.20 ± 7.24 years, and the average course of disease was 25.40 ± 14.94 months. The average age of healthy controls was 25.10 ± 2.96 years. The body mass index of T1DM patients and control subjects were 21.26 ± 2.71 and 20.40 ± 2.24 kg/m^2^, respectively. The detailed description of inclusion and exclusion criteria for T1DM subjects and healthy controls was described in our previous study [15]. This research was approved by the ethics review board of the Second Xiangya Hospital, and all procedures were complied with the ethical principal of Helsinki Declaration. All the participants fully understood the goals and process of the research and provided the written informed consent.

### 2.2. Exosome Isolation and Exosomal circRNA Sequencing

The isolation and characterization of exosomes have been described previously [15]. Briefly, exosomes were isolated by using size exclusion chromatography method with Exosupur® columns (Echobiotech, Beijing, China) from plasma of T1DM patients and control subjects. The transmission electron microscopy (TEM), nanoparticle tracking analysis (NTA), and western blot (WB) were used to validate the isolated fractions. Total exosomal RNA was prepared using miRNeasy Serum/Plasma Advanced Kit (Qiagen, cat. no. 217204), and the concentration as well as purity of RNA were assessed by using the RNA Nano 6000 Assay Kit of the Agilent Bioanalyzer 2100 System (Agilent Technologies, CA, USA). After the library preparation, the exosomal circRNA sequencing was performed by the Illumina Novaseq6000 platform. The raw sequencing data are available in the CNGB Sequencing Archive (CNSA) of China National GeneBank (CNGBdb) repository (https://db.cngb.org/search/project/CNP0002574/).

### 2.3. circRNA Analysis

CircRNAs were predicted using CIRI (circRNA identifier). Annotation of the circRNA-associated genes was performed based on the following databases: Nr (NCBI nonredundant protein sequences), Pfam (protein family), KOG/COG (Cluster of Orthologous Groups of proteins), and Swiss-Prot (http://www.ebi.ac.uk/swissprot/). The raw junction reads for all the samples were normalized to TPM (transcripts per million) by the number of total circRNA mapped reads, and the circRNA junction reads region length was set to 300. Sequence data analyses were mainly performed using R v3.5.1. Pie chart, bar chart, and Circos diagram were generated to visualize the overall expression of circRNAs using R package pie, ggplot2, and Circos software (v0.69), respectively. We used the Database for Annotation, Visualization and Integrated Discovery (DAVID) bioinformatics web server (https://david.ncifhttp://crf.gov/tools.jsp/) as a tool to explore the potential function of these differentially expressed circRNA-associated genes by performing Gene Ontology (GO; http://www.geneontology.org/) and Kyoto Encyclopedia of Genes and Genomes (KEGG) pathway enrichment analyses. GO enrichment analysis was implemented by the topGO R packages. We used KOBAS software to test the statistical enrichment of differential expression genes in KEGG pathways [19].

### 2.4. circRNA-miRNA-mRNA Network Analysis

CircRNA could regulate gene expression by serving as a molecular sponge to sequester the miRNA. To further reveal the potential circRNA-miRNA-mRNA interactions, RNAhybrid (v2.1.1) and miRanda (v3.3a) were used to predict the miRNA-circRNA targeting relationships. MultiMiR package was used for miRNA-mRNA interaction analysis. We used Cytoscape to visualize the obtained circRNA-miRNA-mRNA interaction network. Based on the miRNA targeting results obtained by MultiMiR, GO and KEGG enrichment analyses were carried out using topGO R package and KOBAS software, respectively, to obtain the enrichment analysis results of identified miRNA targeting differentially expressed circRNA.

### 2.5. Statistical Analysis

Results were expressed as the mean ± SD (standard deviation). The significantly differentially expressed circRNAs between T1DM patients and control subjects was performed using the Mann–Whitney *U* test with TPM > 10, cutoff *P* value < 0.05, and fold change > 1.5. *P* value less than 0.05 was viewed as statistically significant. Hierarchical clustering, heatmap, volcano diagram, and M-versus-A (MA) plot was generated to visualize the differentially expressed circRNAs using R package pheatmap and ggplot2.

## 3. Results

### 3.1. Validation of the Plasma-Derived Exosomes

**Figure 1:**
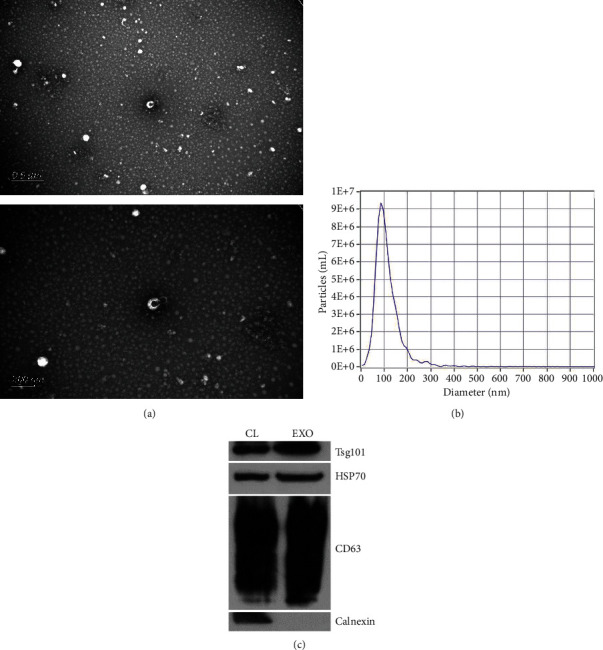
Characterization of exosomes. Transmission electron microscopy images of exosomes isolated from plasma (a). The size distribution of exosomes indicated by nanoparticle tracking analysis (b). Western blot analysis of Alix, Tsg101, CD63, and Calnexin in plasma-derived exosomes (c).

The TEM and NTA indicated that the exosomes showed typic vesicle structure and the diameter range 30-200 nm (median, 111.9 nm) (Figures 1(a) and 1(b)), consistent with characteristics of exosomes described previously. WB analysis indicated exosomal markers Alix, Tsg101, and CD63 were enriched in isolated fraction, while the Calnexin, the negative marker for exosomes, was absent (Figure 1(c)). In short, the exosomes we isolated were well prepared and with relatively high purity.

### 3.2. An Overview of the Sequencing Data

**Figure 2:**
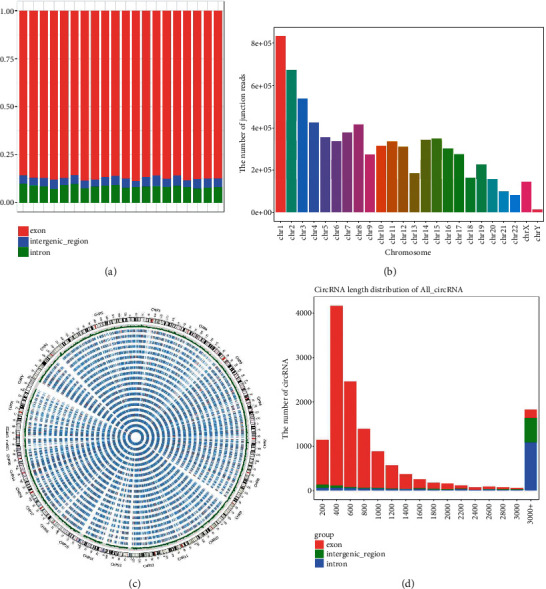
The overview of the exosomal circRNA sequencing data. Pie chart of circRNA type (a). Read distribution of circRNAs on different chromosomes (b). The Circos diagram of the expression level of each sample (c). Length distribution of cirRNAs (d).

20 samples including 10 T1DM patients and 10 age-matched (*P* = 0.97) and sex-matched (*P* = 0.36) healthy controls have been sequenced. Totally, 13813 exosomal circRNAs including 10542 annotated and 3271 novel circRNAs have been identified using Illumina Novaseq6000 sequencing platform. Among them, the most type of circRNA was from exon, followed by from intron and intergenic region (Figure 2(a)). The identified circRNAs was mostly located on chromosome 1 and chromosome 2 and extensively distributed in the human genome (Figure 2(b)). In addition, the Circos diagram was used to display the distribution and expression of circRNAs (Figure 2(c)). The length distribution of circRNA was analyzed, and the length ranging 400-600 bp accounted for the most proportion (Figure 2(d)).

### 3.3. Differentially Expressed Exosomal circRNAs

**Figure 3:**
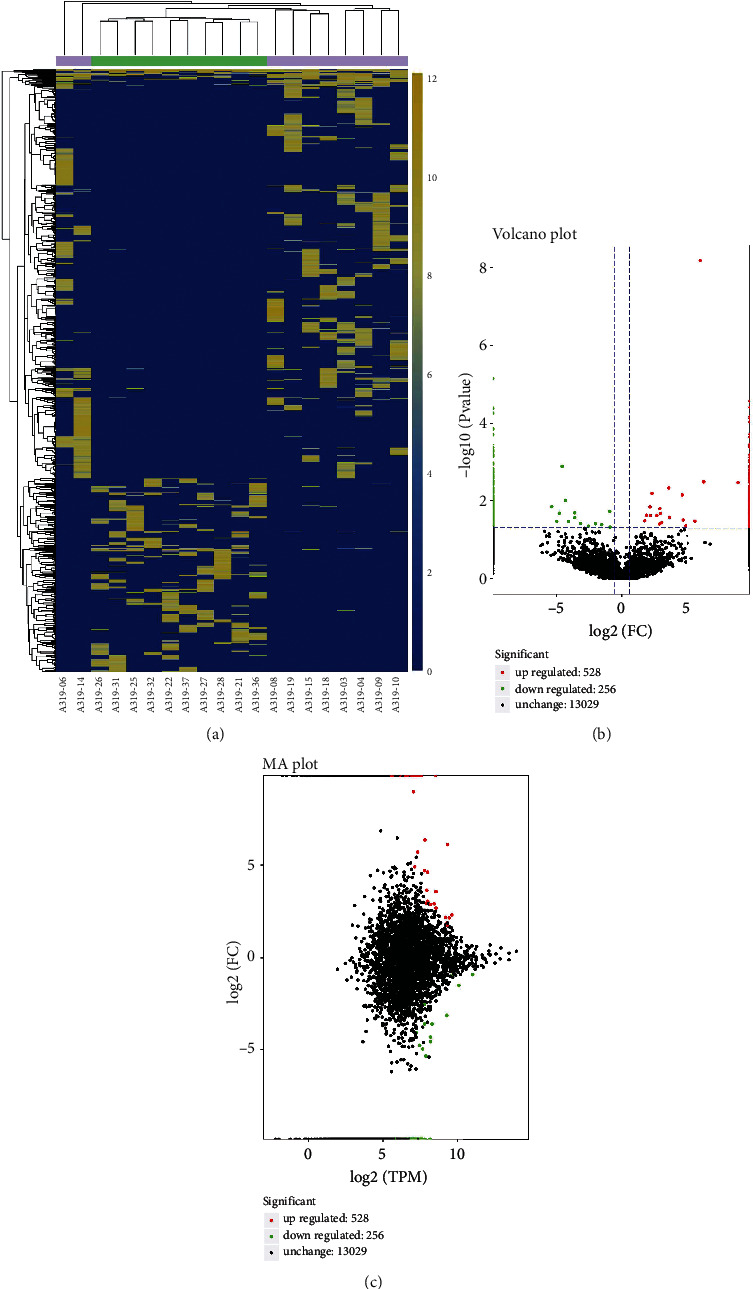
Differential exosomal circRNA profiles in T1DM patients and healthy controls. Heatmap of the expression levels of differentially expressed exosomal circRNAs (a). Volcano plot of the identified circRNAs (b). The MA diagram of the overall distribution of exosomal circRNAs (c).

According to the expression profile, total of 784 differentially expressed circRNAs in which 528 upregulated and 256 downregulated were detected (Supplementary Table 1). The heatmap, volcano diagram, and MA plot were shown in Figure 3 to exhibit the differential profiles of exosomal circRNA in T1DM. In sum, these analyses indicated the expression of exosomal circRNAs were distinguishable between T1DM patients and control subjects.

### 3.4. Bioinformatics Analysis of Exosomal circRNA

To investigate their potential biological function, we performed GO analysis regarding the parental gene of identified exosomal circRNAs. The GO terms included three categories, biological process (BP), cellular component (CC), and molecular function (MF). The parental genes of circRNAs were enriched and analyzed and the enriched terms were shown by topGO direct acyclic graph which could display their hierarchical relationships (Supplementary Figure 1–3). The most enriched terms were establishment of cell polarity (GO:0030010) (BP), cytosol (GO:0005829) (CC), and identical protein binding (GO:0042802) (MF), respectively. In addition, we used COG (Cluster of Orthologous Groups of proteins) database which was constructed based on the phylogenetic relationships of bacteria, algae, and eukaryotes to orthologous classify the gene products, and the results indicated that “general function prediction only” accounted for the most proportion among the function class (Supplementary Figure 4).

KEGG database was the main public database about the metabolic pathways. We classified the annotation results of source gene of circRNA according to the pathway types in KEGG, and the term endocytosis (ko04144) in cellular process was the pathway with most genes (Supplementary Figure 5). We also performed enriched pathway analysis, and the most enriched pathway among these circRNAs was involved in the lysine degradation (ko00310) (Supplementary Figure 6).

### 3.5. Bioinformatics Analysis of Differentially Expressed circRNAs

**Figure 4:**
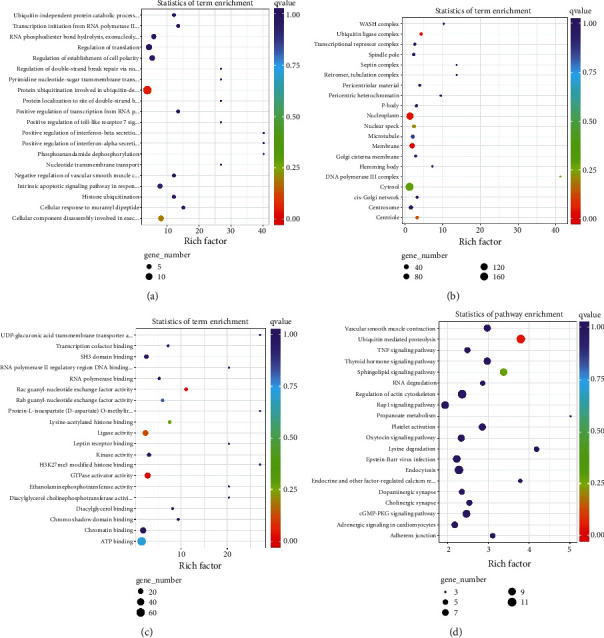
Biological analysis of parental genes of differentially expressed circRNAs, Top 20 Gene Ontology terms for biological processes (a), cellular component (b), and molecular function (c). Top 20 terms of Kyoto Encyclopedia of Genes and Genomes pathway analysis (d).

To further explore the biological function of exosomal circRNAs in T1DM, we carried out the GO and KEGG pathway analyses focusing on the identified differentially expressed circRNAs. The predicted GO terms with the strongest enrichment scores are protein ubiquitination involved in ubiquitin-dependent protein catabolic protein (GO:0042787) (BP), membrane (GO:0016020) (CC), and GTPase activator activity (GO:0005096) (MF) (Figures 4(a)–4(c)). Meanwhile, the most significantly enriched KEGG pathway is involved in the ubiquitin-mediated proteolysis (ko04120) (Figure 4(d)).

### 3.6. Prediction of miRNA Targeted to Differentially Expressed circRNA

**Figure 5:**
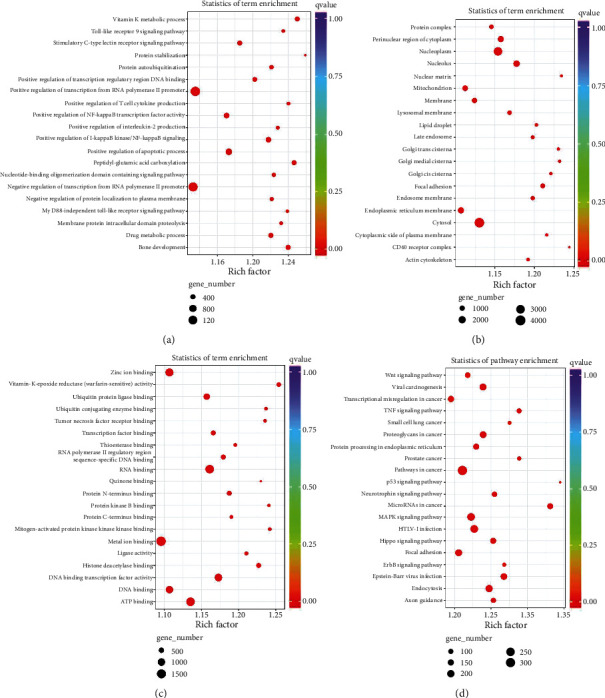
Bioinformatics analysis of identified miRNAs targeted at differentially expressed circRNAs. Top 20 Gene Ontology terms for biological processes (a), cellular component (b), and molecular function (c). Top 20 terms of Kyoto Encyclopedia of Genes and Genomes pathway analysis (d).

**Figure 6:**
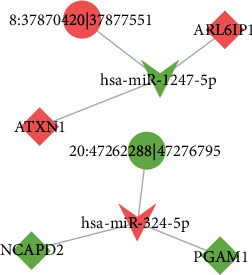
Construction of the circRNA-miRNA-mRNA competing endogenous RNA regulatory network. Diamond indicates diabetes-related gene, tip triangle indicates diabetes-related miRNA, circle indicates diabetes-related circRNA, red color indicated upregulation, and green color indicates downregulation. 8 : 37870420|37877551 (hsa_circ0005630); 20 : 47262288|47276795 (hsa_circ0007026).

The miRNA-targeting prediction method were performed on differentially expressed circRNA, and their potential function could be clarified by functional annotation of identified miRNA. We performed GO and KEGG pathway enrichment analyses regarding identified miRNAs targeting to differentially expressed circRNAs. The most enriched terms are positive regulation from RNA polymerase II promoter (GO:0045944) (BP), cytosol (GO:0005829) (CC), RNA binding (GO:0003700) (MF), and pathway in cancer (ko05200) (KEGG), respectively (Figure 5). Because circRNA could regulate the gene expression by interacting with miRNA, we also constructed the circRNA-miRNA-gene network diagram and the results indicated that the signaling hsa_circ0005630-miR-1247-5p-ATXN1/ARL6IP1 and hsa_circ0007026-miR-324-5p-NCAPD2/PGAM1 might be involved in the progression of T1DM (Figure 6).

## 4. Discussions

At present, the exact pathogenic mechanisms of T1DM have not been fully revealed. However, mounting evidence has suggested both exosome and circRNA were involved in the initiation and progression of T1DM [6, 7, 10, 20]. In the previous study, we have identified the exosomal lncRNA and mRNA expression profiles of T1DM and our results indicated the exosomal lncRNAs and mRNAs might be associated with the development of T1DM [14, 15]. Here, we reported the expression profiles of plasms-derived exosomal circRNA and our results might provide novel insights into the etiopathogenesis of T1DM.

A total of 13813 exosomal circRNAs have been detected. Among them, the most type of circRNA was exonic, which was in accordance with previous studies [6, 21]. These circRNAs were mainly from chromosome 1 and chromosome 2; meanwhile, it is spread around the whole genome, indicating that each chromosome might contribute to the dysregulation of circRNA, thus taking part in the pathogenesis of T1DM.

In the current study, 784 significantly altered exosomal circRNAs in T1DM patients have been identified, with 528 and 256 being up- and downregulated, respectively. This result indicated that the expression profiles of exosomal circRNA derived from plasma of T1DM patients remarkably differ from that in healthy controls. It points out the biomarker use of exosomal circRNA in the T1DM diagnosis. However, the sample size of this study is relatively small. Future research needs to include a larger sample size for verification the biomarker use of exosomal circRNA in clinics. Previous studies have also reported the differential profile of circRNAs in T1DM [6, 18]. Li et al. screened plasma circRNA expression profiles in newly onset T1DM using microarray and identified 68 differentially expressed circRNA [18]. Luo et al. identified the circRNA expression profiles in the peripheral blood of T1DM patients and found 93 differentially expressed circular transcripts [6]. These results highlighted the biomarker potential of circRNAs in T1DM. Besides, exosomal circRNAs have more advantages as biomarker. For instance, they are more stable because the lipid bilayer-enclosed structures protect the RNA degradation [9]. In addition, the release and content of exosomes are strictly regulated by physical and pathological stimuli, thus reflecting the disease state and stage [7].

Next, we explored the potential biological function of identified differentially expressed exosomal circRNAs using multiple bioinformatics analysis. The GO and KEGG pathway analyses enriched terms such as ubiquitin-dependent protein catabolic protein (GO:0042787) (BP), membrane (GO:0016020) (CC), GTPase activator activity (GO:0005096) (MF), and ubiquitin-mediated proteolysis (ko04120) (KEGG), and some terms were involved in the diabetes or its complications [22, 23]. The results indicated that exosomal circRNAs might be associated with T1DM progression via multiple mechanisms. Given that circRNAs could function as sponges to sequester certain miRNAs, we predicted complimentary miRNAs targeting to identified differentially expressed circRNAs and constructed the circRNA-miRNA-gene networks. We identified the signaling, hsa_circ0005630-miR-1247-5p-ATXN1/ARL6IP1 and hsa_circ0007026-miR-324-5p-NCAPD2/PGAM1, might take part in the development of T1DM. However, the results need to be interpreted with caution because further *in vitro* and *in vivo* studies are necessary to validate their exact role in T1DM. Previous studies have demonstrated the circRNA-miRNA-gene signaling pathways were related to T1DM progression. For instance, circPPM1F could regulate M1 macrophage activation via circPPM1F-HuR-PPM1F-NF-*κ*B axis and overexpression of circPPM1F could promote pancreatic islet injury through enhancing M1 macrophage activation [20]. Besides, a recent study has indicated that circ_0060450 could negatively modulate type I interferon-induced inflammation by sponging miR-199a-5p in T1DM [24].

Mounting evidence has highlighted the importance of exosomal circRNAs in multiple diseases, especially in cancers. For instance, it has been indicated that exosomal circRNA-100338 could accelerate hepatocellular carcinoma metastasis through enhancing invasiveness and angiogenesis [25]. In addition, an exosomal circPACRGL-miR-142-3p/miR-506-3p-TGF-*β*1 axis has been suggested to promote colorectal cancer progression [26]. Furthermore, exosomal circSHKBP1 has been demonstrated to promote gastric cancer progression via regulating the miR-582-3p/HUR/VEGF pathway and suppressing HSP90 degradation [27]. Other studies have also shown that exosomal circRNAs were related to the occurrence and development of autoimmune diseases. Sun et al. characterized the plasma exosomal circRNA expression profiles in Grave's disease and identified multiple pathways associated with immune system activation [28]. Interestingly, some exosomal circRNAs have been indicated to exert therapeutic effect on autoimmune diseases. For instance, synovial MSC-derived exosomal circRNAs have therapeutic potential on rheumatoid arthritis (RA) via targeting circEDIL3/miR-485-3p/PIAS3/STAT3/VEGF function module [29]. Furthermore, MSC-derived exosomal circFBXW7 could alleviate cell proliferation, migration, and inflammation of fibroblast-like synoviocytes via modulating miR-216a-3p/HDAC4 in RA [30].

There are several disadvantages of our study. First, the sample size was relatively small; future study should include more participants and validate the expression profiles of identified exosomal circRNA by qRT-PCR. Second, *in vitro* and *in vivo* studies were needed to elucidate the exact function of exosomal circRNA.

Both exosome and circRNA have been shown to play an important role in T1DM pathogenesis. However, there is lack of relevant research on the exact role of exosomal circRNA in T1DM development. In this study, we reported the expression profiles of exosomal circRNA in T1DM for the first time and explored their potential biological function. Our results laid foundation for the possible biomarker and therapeutic use for exosomal circRNAs in T1DM.

## Data Availability

The datasets generated and/or analyzed during the current study are available in the CNGB Sequencing Archive (CNSA) of China National GeneBank (CNGBdb) repository, accession number CNP0002574 (https://db.cngb.org/search/project/CNP0002574/).

## Supplementary Materials

Supplementary 1 Supplementary Figure 1: Gene Ontology enrichment analysis (biological process) of parental genes of identified circRNAs.

Supplementary 2 Supplementary Figure 2: Gene Ontology enrichment analysis (cellular component) of parental genes of identified circRNAs.

Supplementary 3 Supplementary Figure 3: Gene Ontology enrichment analysis (molecular function) of parental genes of identified circRNAs.

Supplementary 4 Supplementary Figure 4: the Cluster of Orthologous Groups of proteins (COG) function classification of parental genes of circRNAs.

Supplementary 5 Supplementary Figure 5: the Kyoto Encyclopedia of Genes and Genomes (KEGG) pathway analysis of parental genes of circRNAs. The annotation results were classified according to the pathway types in KEGG.

Supplementary 6 Supplementary Figure 6: the Kyoto Encyclopedia of Genes and Genomes (KEGG) enrichment analysis. The top 20 pathways with the lowest significant *Q* value were shown. The closer to the upper right corner of the figure, the greater the reference value.

Supplementary 7 Supplementary Table 1: the detailed information of the differentially expressed exosomal circRNAs.
