# Supplementary figures and images for: Differential Expression and Bioinformatics Analysis of Plasma-Derived Exosomal circRNA in Type 1 Diabetes Mellitus
Source: J Immunol Res. 2022 Oct 27;2022:3625052. doi: 10.1155/2022/3625052 (PMC9634467; doi:10.1155/2022/3625052)

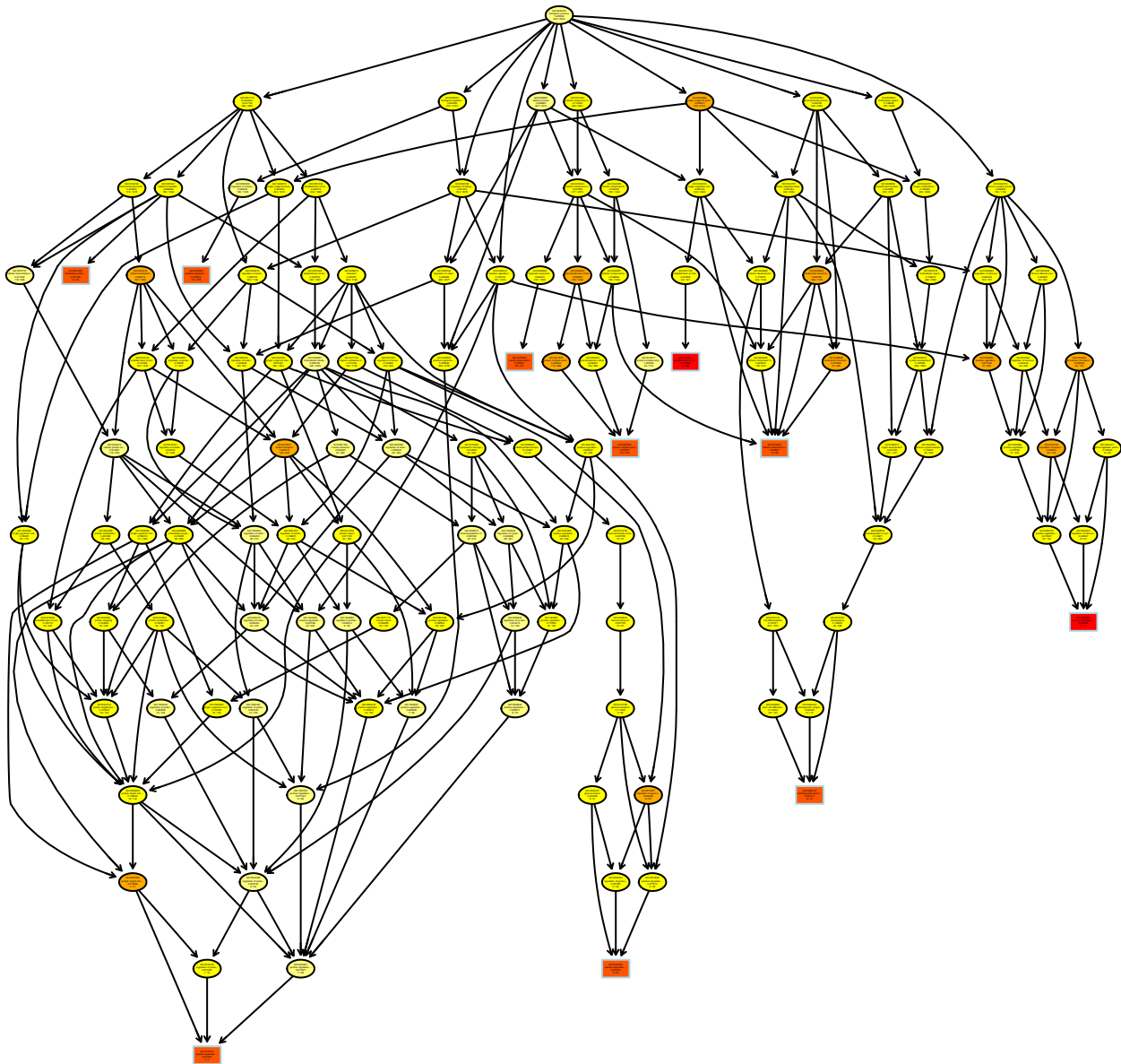

Supplement: Supplementary 1 — Supplementary Figure 1: Gene Ontology enrichment analysis (biological process) of parental genes of identified circRNAs. [file 3625052.f1.pdf]

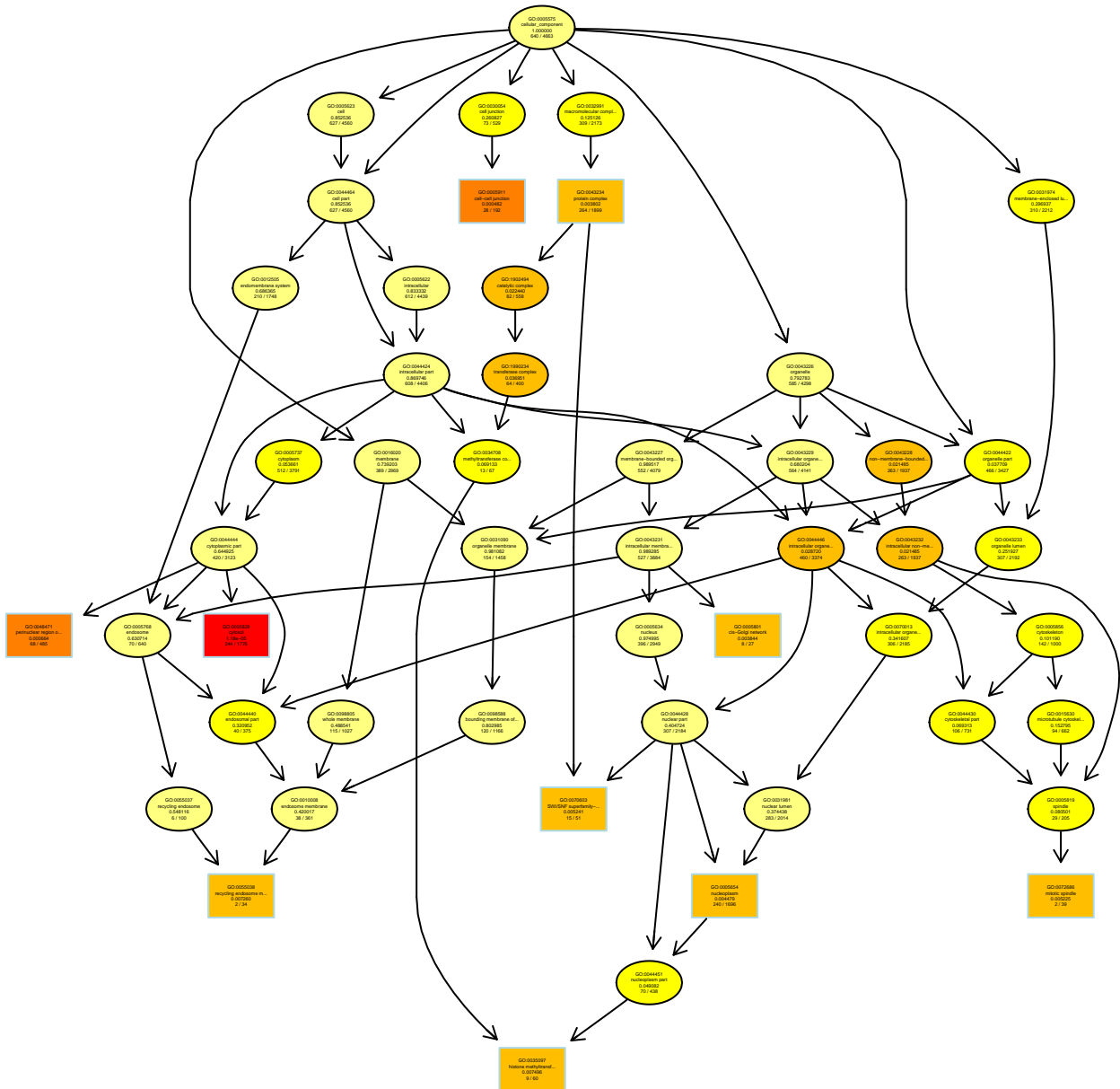

Supplement: Supplementary 2 — Supplementary Figure 2: Gene Ontology enrichment analysis (cellular component) of parental genes of identified circRNAs. [file 3625052.f2.pdf]

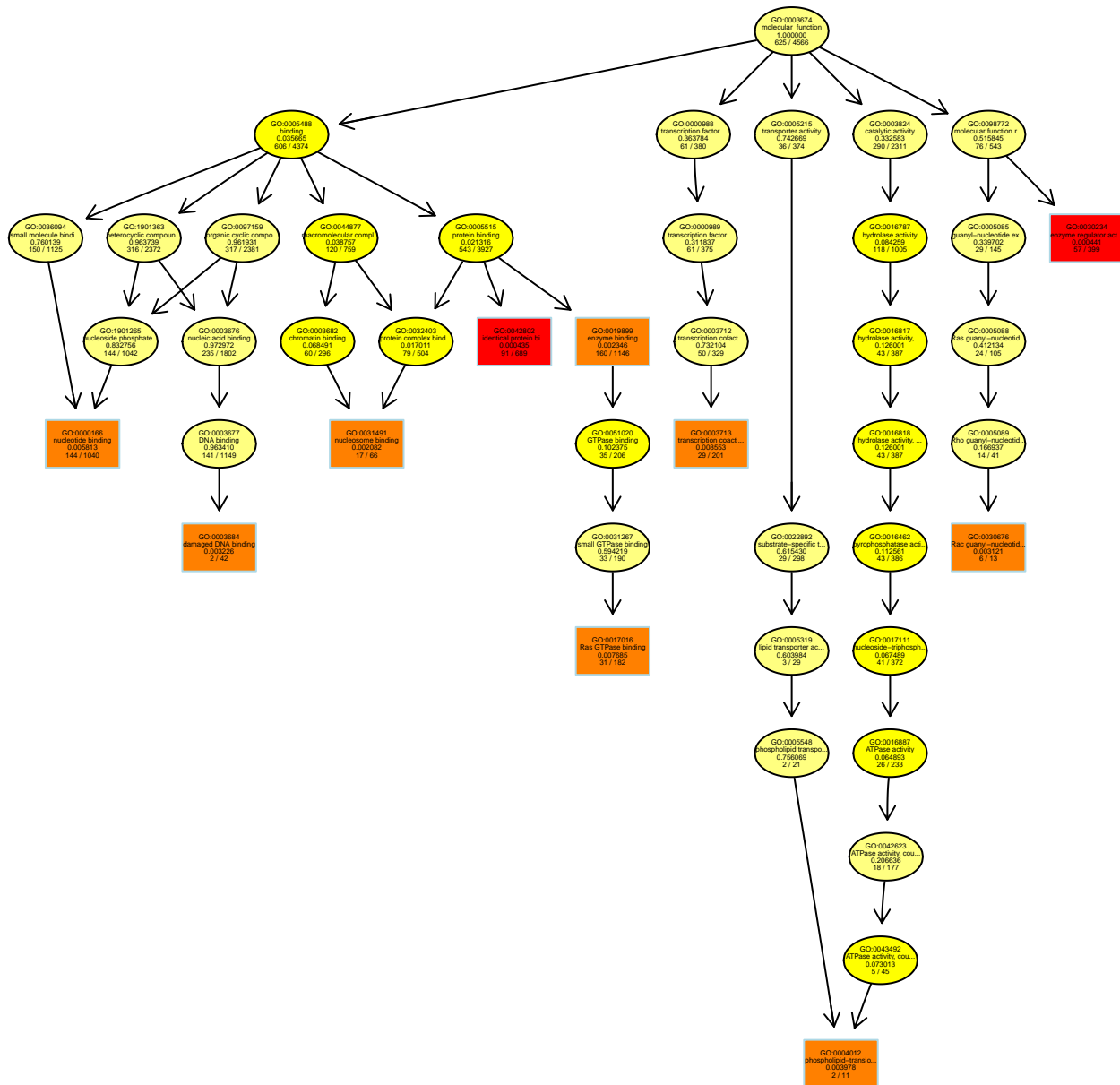

Supplement: Supplementary 3 — Supplementary Figure 3: Gene Ontology enrichment analysis (molecular function) of parental genes of identified circRNAs. [file 3625052.f3.pdf]

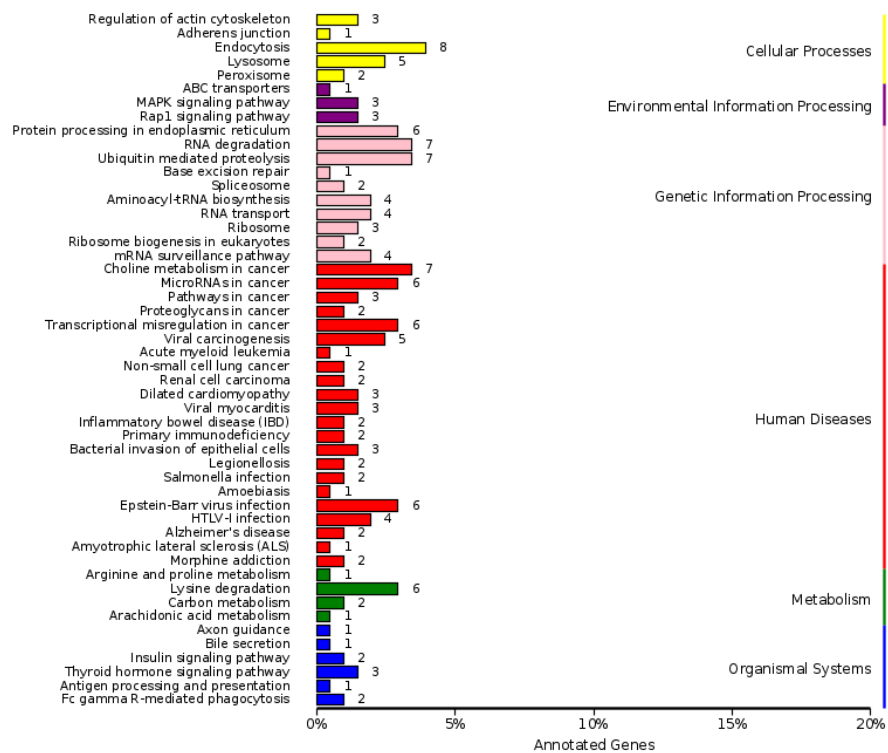

Supplement: Supplementary 5 — Supplementary Figure 5: the Kyoto Encyclopedia of Genes and Genomes (KEGG) pathway analysis of parental genes of circRNAs. The annotation results were classified according to the pathway types in KEGG. [file 3625052.f5.pdf]

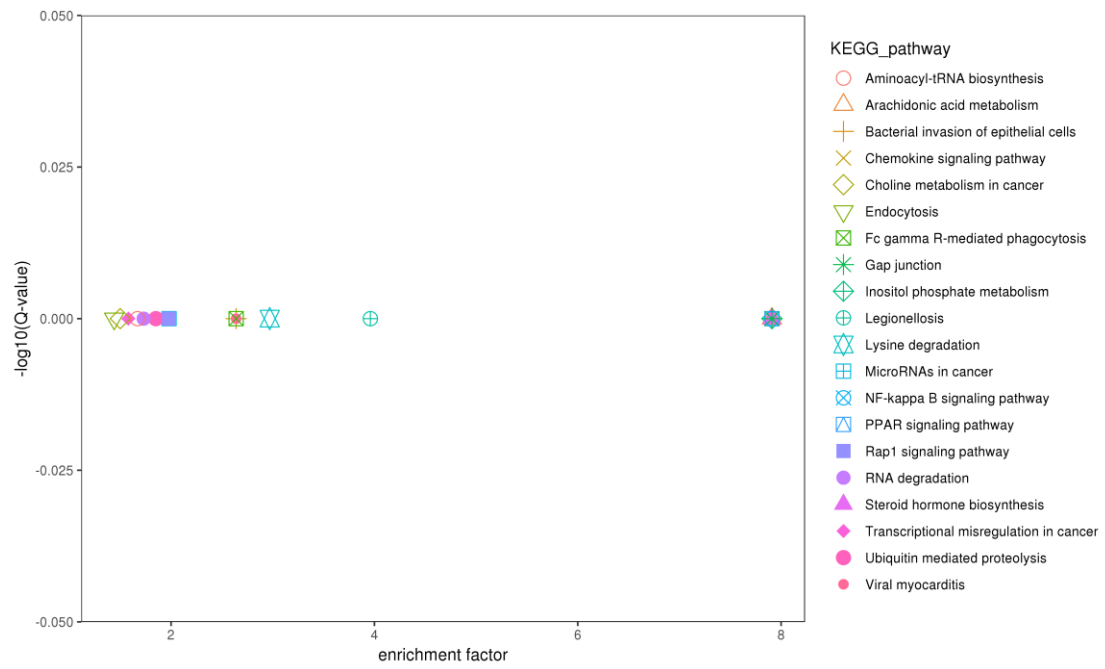

Supplement: Supplementary 6 — Supplementary Figure 6: the Kyoto Encyclopedia of Genes and Genomes (KEGG) enrichment analysis. The top 20 pathways with the lowest significant Q value were shown. The closer to the upper right corner of the figure, the greater the reference value. [file 3625052.f6.pdf]
