# Supplementary material for: Differential Expression and Bioinformatics Analysis of Plasma-Derived Exosomal circRNA in Type 1 Diabetes Mellitus
Source: J Immunol Res. 2022 Oct 27;2022:3625052. doi: 10.1155/2022/3625052 (PMC9634467; doi:10.1155/2022/3625052)
Supplement: Supplementary 4 — Supplementary Figure 4: the Cluster of Orthologous Groups of proteins (COG) function classification of parental genes of circRNAs. [file 3625052.f4.pdf]

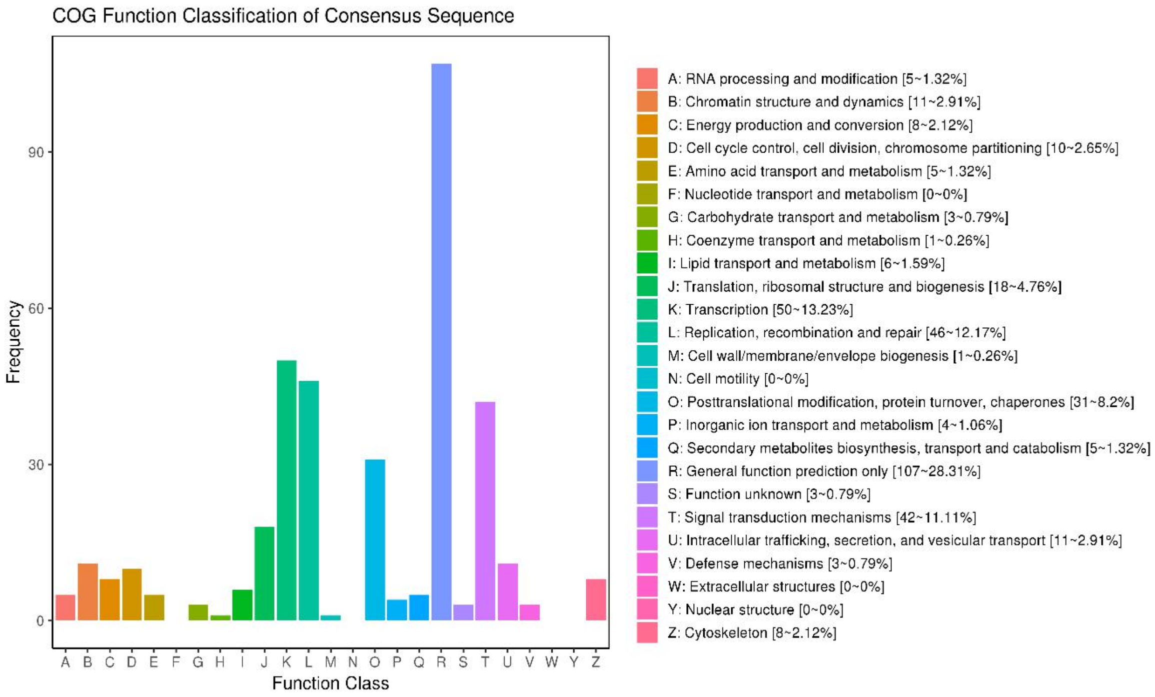
COG Function Classification of Consensus Sequence
Frequency
0
30
60
90
A B C D E F G H I J K L M N O P Q R S T U V W Y Z
Function Class
A: RNA processing and modification [5~1.32%]
B: Chromatin structure and dynamics [11~2.91%]
C: Energy production and conversion [8~2.12%]
D: Cell cycle control, cell division, chromosome partitioning [10~2.65%]
E: Amino acid transport and metabolism [5~1.32%]
F: Nucleotide transport and metabolism [0~0%]
G: Carbohydrate transport and metabolism [3~0.79%]
H: Coenzyme transport and metabolism [1~0.26%]
I: Lipid transport and metabolism [6~1.59%]
J: Translation, ribosomal structure and biogenesis [18~4.76%]
K: Transcription [50~13.23%]
L: Replication, recombination and repair [46~12.17%]
M: Cell wall/membrane/envelope biogenesis [1~0.26%]
N: Cell motility [0~0%]
O: Posttranslational modification, protein turnover, chaperones [31~8.2%]
P: Inorganic ion transport and metabolism [4~1.06%]
Q: Secondary metabolites biosynthesis, transport and catabolism [5~1.32%]
R: General function prediction only [107~28.31%]
S: Function unknown [3~0.79%]
T: Signal transduction mechanisms [42~11.11%]
U: Intracellular trafficking, secretion, and vesicular transport [11~2.91%]
V: Defense mechanisms [3~0.79%]
W: Extracellular structures [0~0%]
Y: Nuclear structure [0~0%]
Z: Cytoskeleton [8~2.12%]

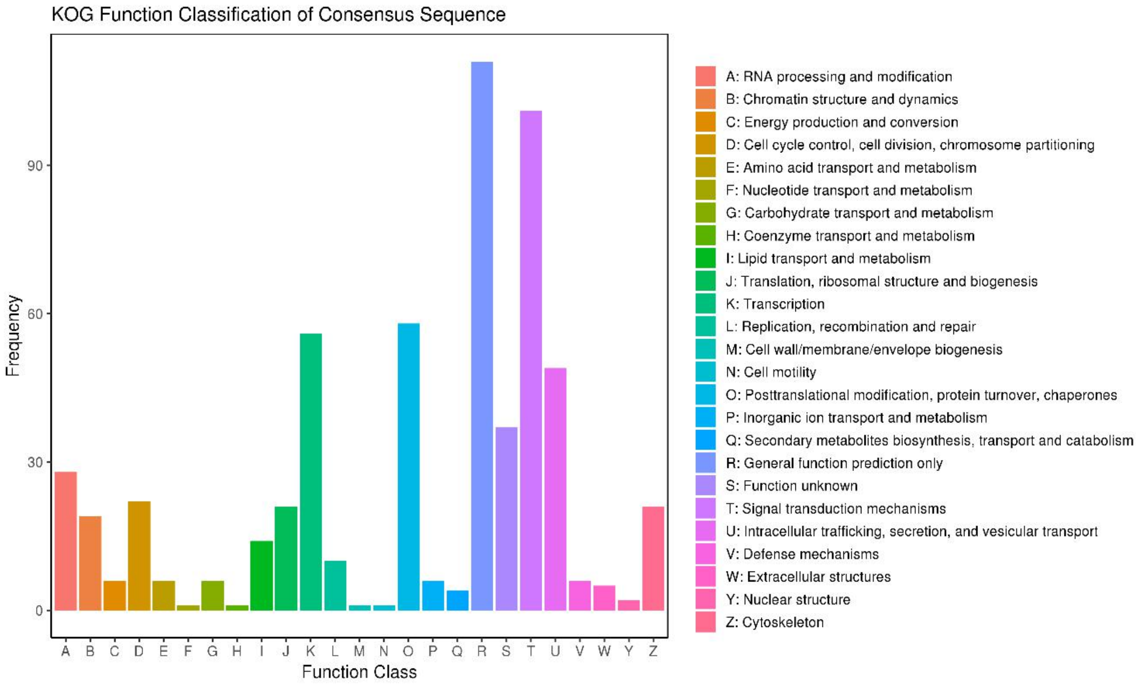
KOG Function Classification of Consensus Sequence
Frequency
0
30
60
90
A B C D E F G H I J K L M N O P Q R S T U V W Y Z
Function Class
A: RNA processing and modification
B: Chromatin structure and dynamics
C: Energy production and conversion
D: Cell cycle control, cell division, chromosome partitioning
E: Amino acid transport and metabolism
F: Nucleotide transport and metabolism
G: Carbohydrate transport and metabolism
H: Coenzyme transport and metabolism
I: Lipid transport and metabolism
J: Translation, ribosomal structure and biogenesis
K: Transcription
L: Replication, recombination and repair
M: Cell wall/membrane/envelope biogenesis
N: Cell motility
O: Posttranslational modification, protein turnover, chaperones
P: Inorganic ion transport and metabolism
Q: Secondary metabolites biosynthesis, transport and catabolism
R: General function prediction only
S: Function unknown
T: Signal transduction mechanisms
U: Intracellular trafficking, secretion, and vesicular transport
V: Defense mechanisms
W: Extracellular structures
Y: Nuclear structure
Z: Cytoskeleton
