# Supplementary material for: Differential Expression and Bioinformatics Analysis of Plasma-Derived Exosomal circRNA in Type 1 Diabetes Mellitus
Source: J Immunol Res. 2022 Oct 27;2022:3625052. doi: 10.1155/2022/3625052 (PMC9634467; doi:10.1155/2022/3625052)
Supplement: Supplementary 7 — Supplementary Table 1: the detailed information of the differentially expressed exosomal circRNAs. [file 3625052.f7.pdf]

#ID	P-Value	FDR	log2FC	Regulated
1:1712264|1716478	0.000659727	0.232918694	Inf	up
1:1804419|1815862	0.038544646	0.232918694	#NAME?	down
1:1804419|1817875	0.008220523	0.232918694	Inf	up
1:1804419|1839238	3.97E-05	0.091475138	Inf	up
1:10095461|10105744	0.015866561	0.232918694	Inf	up
1:10291082|10297246	0.005882573	0.232918694	#NAME?	down
1:12001401|12002103	0.037278	0.232918694	3.05602796	up
1:15536475|15536814	0.021604355	0.232918694	Inf	up
1:21002713|21111383	0.047568274	0.232918694	Inf	up
1:23055069|23069151	0.032209314	0.232918694	Inf	up
1:24649971|24651612	0.045775647	0.232918694	Inf	up
1:28048383|28058094	0.02401887	0.232918694	Inf	up
1:28476107|28480604	0.012173763	0.232918694	Inf	up
1:28580560|28581229	0.034063652	0.232918694	Inf	up
1:28987431|28997319	0.04989836	0.232918694	Inf	up
1:29148080|29149226	0.024902481	0.232918694	#NAME?	down
1:30992390|30995220	0.041968881	0.232918694	-1.5286904	down
1:31346640|31348974	0.025806669	0.232918694	Inf	up
1:33294937|33295305	0.023772867	0.232918694	1.89007288	up
1:35392212|35399576	0.012668496	0.232918694	Inf	up
1:35745451|35746992	0.039700765	0.232918694	Inf	up
1:37987573|37987836	0.010981889	0.232918694	#NAME?	down
1:42265115|42323827	0.043835573	0.232918694	#NAME?	down
1:51840226|51840514	0.042052131	0.232918694	Inf	up
1:61109778|61112343	0.047562715	0.232918694	Inf	up
1:61111371|61112343	0.02409574	0.232918694	Inf	up
1:61404104|61406727	0.049515606	0.232918694	Inf	up
1:65364635|65366196	0.033015384	0.232918694	5.7023042	up
1:66958059|66959743	0.021582982	0.232918694	#NAME?	down
1:75732644|75750546	0.024903621	0.232918694	#NAME?	down
1:75745806|75750546	0.014825343	0.232918694	#NAME?	down
1:77916055|77918273	0.001974102	0.232918694	Inf	up
1:88770352|88771879	0.027111395	0.232918694	Inf	up
1:100136882|100137085	0.014847246	0.232918694	#NAME?	down
1:108752257|108760085	0.029196265	0.232918694	Inf	up
1:110891392|110892533	0.000718435	0.232918694	#NAME?	down
1:111147642|111174963	0.004566966	0.232918694	Inf	up
1:112591525|112616895	0.010998361	0.232918694	#NAME?	down
1:112653598|112667145	0.014127249	0.232918694	Inf	up
1:113825142|113834439	0.008764444	0.232918694	Inf	up
1:150226608|150229237	0.024528871	0.232918694	Inf	up
1:151117392|151118139	0.02527943	0.232918694	Inf	up
1:151638888|151668635	0.046794375	0.232918694	Inf	up
1:153811731|153813452	0.018204292	0.232918694	Inf	up
1:155661307|155777820	0.04696687	0.232918694	#NAME?	down
1:155717006|155726019	0.047247375	0.232918694	Inf	up
1:155813634|155814449	0.038216939	0.232918694	Inf	up
1:156320839|156334918	0.02959139	0.232918694	Inf	up
1:160624096|160634897	0.020318886	0.232918694	#NAME?	down
1:171523221|171532961	0.03438895	0.232918694	-4.956657	down

1:172042009|172131288	0.034692356	0.232918694	Inf	up
1:172551512|172579267	0.000765182	0.232918694	Inf	up
1:174272414|174287770	0.046833081	0.232918694	Inf	up
1:174272414|174305127	0.001890568	0.232918694	Inf	up
1:174304986|174371072	0.008664354	0.232918694	#NAME?	down
1:180084018|180087717	0.045333363	0.232918694	#NAME?	down
1:185202654|185222036	0.011862867	0.232918694	Inf	up
1:193203795|193212477	0.007673997	0.232918694	Inf	up
1:205268950|205269884	0.043434283	0.232918694	Inf	up
1:205727700|205729621	0.003237604	0.232918694	#NAME?	down
1:207694353|207740418	0.010993041	0.232918694	Inf	up
1:209830108|209831043	0.00193635	0.232918694	#NAME?	down
1:219193082|219218712	0.045158895	0.232918694	Inf	up
1:220102129|220102777	0.017083108	0.232918694	#NAME?	down
1:220212887|220213979	0.008570858	0.232918694	Inf	up
1:222823884|222835215	0.035052073	0.232918694	Inf	up
1:224267004|224268133	0.048816449	0.232918694	Inf	up
1:224317694|224326464	0.006516432	0.232918694	#NAME?	down
1:224365879|224371423	0.030490816	0.232918694	Inf	up
1:224952670|225007544	0.00860133	0.232918694	#NAME?	down
1:235800320|235801097	0.017447766	0.232918694	#NAME?	down
1:243304713|243330692	0.041720615	0.232918694	#NAME?	down
1:243415702|243426558	0.004757039	0.232918694	Inf	up
1:243637611|243695716	0.003120735	0.232918694	Inf	up
10:263204|263658	0.02789972	0.232918694	Inf	up
10:1056348|1072293	0.027230449	0.232918694	#NAME?	down
10:4830675|4847299	0.030966116	0.232918694	Inf	up
10:4830675|4909606	0.001171049	0.232918694	#NAME?	down
10:4929656|4933330	0.011941029	0.232918694	Inf	up
10:5699525|5699737	0.028874322	0.232918694	Inf	up
10:5956379|5960567	0.048897545	0.232918694	Inf	up
10:11936545|11952249	0.01052685	0.232918694	Inf	up
10:13663453|13670528	0.049061402	0.232918694	Inf	up
10:14928016|14935564	0.025191158	0.232918694	Inf	up
10:15833630|15847943	0.016283299	0.232918694	2.9155279	up
10:17688027|17695241	0.049933857	0.232918694	Inf	up
10:20957731|20961779	0.014240517	0.232918694	#NAME?	down
10:24380869|24384423	0.048948141	0.232918694	#NAME?	down
10:28595733|28608431	0.006738517	0.232918694	Inf	up
10:30026103|30029866	0.000132191	0.160448253	#NAME?	down
10:31355144|31387798	0.011878899	0.232918694	#NAME?	down
10:31355147|31387266	0.018527407	0.232918694	Inf	up
10:31461037|31502509	0.033855869	0.232918694	Inf	up
10:32034690|32037607	0.001993803	0.232918694	Inf	up
10:35013699|35031619	0.009493095	0.232918694	Inf	up
10:43154897|43158931	0.036790044	0.232918694	Inf	up
10:50460673|50519921	0.015220246	0.232918694	Inf	up
10:50519831|50598823	0.004722393	0.232918694	Inf	up
10:68363590|68364113	0.010213164	0.232918694	#NAME?	down
10:68737845|68747566	0.045708856	0.232918694	#NAME?	down
10:74070670|74074903	0.001394798	0.232918694	Inf	up

10:79118915|79162133	0.045472857	0.232918694	Inf	up
10:86443275|86446449	0.041813095	0.232918694	Inf	up
10:86453220|86460496	0.035790287	0.232918694	Inf	up
10:86517571|86518091	0.01392825	0.232918694	Inf	up
10:87508336|87513221	0.042215515	0.232918694	Inf	up
10:87508336|87521169	0.021499731	0.232918694	2.94820224	up
10:89751346|89762835	0.000195611	0.192998461	Inf	up
10:91994535|92008275	0.030408989	0.232918694	#NAME?	down
10:92531748|92537550	0.046098031	0.232918694	Inf	up
10:96544076|96565426	0.000228476	0.210395709	Inf	up
10:96907265|96907747	0.006632945	0.232918694	2.31465782	up
10:96949008|96952196	0.030301905	0.232918694	Inf	up
10:100937402|100944128	0.049872401	0.232918694	Inf	up
10:101672915|101676436	0.030871882	0.232918694	Inf	up
10:102358039|102359435	0.043622821	0.232918694	#NAME?	down
10:118729295|118730410	0.040741065	0.232918694	Inf	up
10:124408542|124412200	0.031700923	0.232918694	Inf	up
10:126970702|127000307	0.026418771	0.232918694	#NAME?	down
10:126996748|127000307	0.026849027	0.232918694	Inf	up
10:131934458|131947791	0.048205255	0.232918694	Inf	up
10:131943968|131945459	0.014423203	0.232918694	Inf	up
11:581492|587464	0.007204218	0.232918694	Inf	up
11:9989497|10002689	0.047250839	0.232918694	Inf	up
11:14771937|14789242	0.031998956	0.232918694	#NAME?	down
11:16183886|16186955	0.034526074	0.232918694	Inf	up
11:17336960|17337534	0.024917074	0.232918694	#NAME?	down
11:20395803|20397721	0.038966402	0.232918694	#NAME?	down
11:28211062|28233585	0.027372609	0.232918694	Inf	up
11:33338757|33341686	0.023129215	0.232918694	Inf	up
11:34957384|34992379	0.020340081	0.232918694	Inf	up
11:47630555|47631087	0.049537485	0.232918694	Inf	up
11:47752916|47754664	0.002751286	0.232918694	Inf	up
11:57152640|57194765	0.03870907	0.232918694	#NAME?	down
11:61366045|61367998	0.016382753	0.232918694	Inf	up
11:68185781|68190152	0.042242568	0.232918694	Inf	up
11:72984088|73083740	0.012221031	0.232918694	Inf	up
11:74810184|74817714	0.033107244	0.232918694	Inf	up
11:76513386|76528466	0.003100963	0.232918694	#NAME?	down
11:77622500|77629899	0.02080883	0.232918694	Inf	up
11:77691159|77693611	0.030984364	0.232918694	Inf	up
11:85974708|85981228	0.026775825	0.232918694	Inf	up
11:86026292|86031611	0.047380617	0.232918694	Inf	up
11:89400017|89402542	0.007946117	0.232918694	Inf	up
11:95812932|95818904	0.030804887	0.232918694	Inf	up
11:108258986|108267342	0.022722611	0.232918694	#NAME?	down
11:114063211|114187038	0.044813441	0.232918694	Inf	up
11:120431771|120437382	0.006905944	0.232918694	Inf	up
11:128768118|128772985	0.000354908	0.232918694	Inf	up
11:128768118|128782023	0.004823176	0.232918694	3.57835484	up
12:829062|829944	0.024281037	0.232918694	2.18430849	up
12:1180540|1190052	0.003287977	0.232918694	#NAME?	down

12:5799507	5854141	0.033218009	0.232918694	Inf	up
12:6655638	6656798	0.009790826	0.232918694	Inf	up
12:8943405	8943910	0.045757154	0.232918694	Inf	up
12:11034782	11121180	0.027622894	0.232918694	#NAME?	down
12:24213343	24249916	0.015578422	0.232918694	Inf	up
12:25079244	25083493	0.044952562	0.232918694	Inf	up
12:28305649	28391411	0.007620056	0.232918694	#NAME?	down
12:31392267	31392696	0.047576437	0.232918694	Inf	up
12:32708153	32713371	0.036059782	0.232918694	Inf	up
12:32713209	32718763	0.02080789	0.232918694	Inf	up
12:47104113	47108164	0.039794913	0.232918694	-2.0106496	down
12:48074324	48074793	0.004903028	0.232918694	#NAME?	down
12:50430495	50454417	0.009949191	0.232918694	Inf	up
12:50780140	50809589	0.003991632	0.232918694	Inf	up
12:50814010	50814439	0.010648468	0.232918694	#NAME?	down
12:51291591	51299714	0.021448533	0.232918694	#NAME?	down
12:53459272	53468832	0.046369455	0.232918694	Inf	up
12:56670275	56673065	0.023155236	0.232918694	#NAME?	down
12:57395749	57398198	0.023645227	0.232918694	#NAME?	down
12:62484170	62501698	0.042923087	0.232918694	#NAME?	down
12:68713865	68714753	0.031991423	0.232918694	Inf	up
12:68715627	68727389	0.041603427	0.232918694	Inf	up
12:79817394	79822190	0.038149432	0.232918694	Inf	up
12:88148288	88176319	0.046074587	0.232918694	Inf	up
12:89459638	89472275	0.023411706	0.232918694	Inf	up
12:89466770	89472275	0.018898805	0.232918694	Inf	up
12:94169153	94186473	0.001175768	0.232918694	#NAME?	down
12:94220016	94227235	0.0094116	0.232918694	#NAME?	down
12:94254787	94282401	0.044904311	0.232918694	Inf	up
12:95152811	95152993	0.032756296	0.232918694	Inf	up
12:95208843	95211267	0.002381434	0.232918694	#NAME?	down
12:101396399	101405981	0.024035517	0.232918694	#NAME?	down
12:101714090	101716812	0.031364573	0.232918694	Inf	up
12:101897863	101901433	0.044580044	0.232918694	#NAME?	down
12:109101902	109103611	0.025998893	0.232918694	#NAME?	down
12:111482787	111486824	0.009308953	0.232918694	Inf	up
12:111485265	111486824	0.04210154	0.232918694	Inf	up
12:111552280	111555919	0.015490551	0.232918694	Inf	up
12:112178931	112179397	0.01577431	0.232918694	Inf	up
12:112477651	112478015	0.041222404	0.232918694	#NAME?	down
12:116236821	116237705	0.012710151	0.232918694	#NAME?	down
12:120510017	120516788	0.00451414	0.232918694	Inf	up
12:122216488	122224644	0.049348408	0.232918694	Inf	up
12:122327947	122328426	0.021652284	0.232918694	#NAME?	down
12:123160781	123203375	0.029196013	0.232918694	Inf	up
12:123194386	123198334	0.047470269	0.232918694	Inf	up
12:124419957	124430781	0.0097332	0.232918694	#NAME?	down
12:131992173	132006302	0.032242992	0.232918694	Inf	up
13:20596150	20615879	0.04959987	0.232918694	Inf	up
13:21167988	21172681	0.028574202	0.232918694	Inf	up
13:21301419	21301618	0.007323437	0.232918694	#NAME?	down

13:21391722	21425678	0.024890373	0.232918694	Inf	up
13:24498116	24501834	0.047424042	0.232918694	Inf	up
13:28260447	28261458	0.003367622	0.232918694	8.95340672	up
13:30231314	30283791	0.048056577	0.232918694	Inf	up
13:32480590	32527532	0.00212861	0.232918694	#NAME?	down
13:32648754	32652007	0.041469559	0.232918694	#NAME?	down
13:37029765	37051583	0.011226716	0.232918694	#NAME?	down
13:37040405	37051583	0.022744646	0.232918694	Inf	up
13:40559509	40560860	0.049220665	0.232918694	Inf	up
13:40940921	40943925	0.04477609	0.232918694	4.90211361	up
13:41252645	41254620	0.039678067	0.232918694	#NAME?	down
13:42159266	42160136	0.019784399	0.232918694	#NAME?	down
13:45516143	45525051	0.004639415	0.232918694	#NAME?	down
13:48256177	48258236	0.034468869	0.232918694	Inf	up
13:48342599	48349023	0.010768793	0.232918694	Inf	up
13:52417992	52427219	0.000376833	0.232918694	Inf	up
13:52658099	52659249	0.038556434	0.232918694	Inf	up
13:100425633	100449305	0.018046231	0.232918694	Inf	up
13:100668579	100670569	0.040966042	0.232918694	Inf	up
13:109560401	109571158	0.046277279	0.232918694	Inf	up
13:112565111	112571114	0.042268489	0.232918694	Inf	up
14:20395450	20395949	0.029501168	0.232918694	#NAME?	down
14:22906195	22913845	0.041203045	0.232918694	Inf	up
14:24211411	24211829	0.033172103	0.232918694	Inf	up
14:37110827	37123342	0.04382294	0.232918694	Inf	up
14:39154211	39159550	0.028913378	0.232918694	Inf	up
14:49723874	49725769	0.000443991	0.232918694	#NAME?	down
14:50741582	50744365	0.04836423	0.232918694	Inf	up
14:54702062	54702580	0.011055574	0.232918694	Inf	up
14:60796217	60818847	0.006413148	0.232918694	#NAME?	down
14:61443111	61457679	0.013376208	0.232918694	#NAME?	down
14:71413948	71529370	0.003283683	0.232918694	#NAME?	down
14:73107826	73109492	0.043308789	0.232918694	Inf	up
14:91797785	91802421	0.019932988	0.232918694	#NAME?	down
14:92070935	92081061	0.002719984	0.232918694	#NAME?	down
14:92093252	92096838	0.025310268	0.232918694	Inf	up
14:92555751	92577477	0.001870129	0.232918694	#NAME?	down
14:92810254	92811754	0.042950866	0.232918694	Inf	up
14:93016676	93076619	0.041913175	0.232918694	#NAME?	down
14:96560649	96565214	0.005390428	0.232918694	Inf	up
14:103008472	103012188	0.0060332	0.232918694	Inf	up
14:103451918	103462461	0.042532086	0.232918694	#NAME?	down
14:103746373	103754244	0.049795379	0.232918694	Inf	up
15:24975358	24977029	0.011413153	0.232918694	Inf	up
15:28292888	28312874	0.031873355	0.232918694	#NAME?	down
15:40985338	40987974	0.03794069	0.232918694	Inf	up
15:41085369	41087682	0.040365671	0.232918694	#NAME?	down
15:42559339	42563857	0.045617856	0.232918694	Inf	up
15:44595259	44596943	0.022754337	0.232918694	Inf	up
15:44626331	44629388	0.043026447	0.232918694	Inf	up
15:49137150	49139653	0.046717739	0.232918694	Inf	up

15:50300789|50309798	0.002491033	0.232918694	Inf	up
15:56394165|56394834	0.005912174	0.232918694	Inf	up
15:57091789|57095163	0.026396538	0.232918694	Inf	up
15:58912563|58919972	0.049651077	0.232918694	Inf	up
15:58913499|58916999	0.034993869	0.232918694	#NAME?	down
15:59671183|59680308	0.024074286	0.232918694	Inf	up
15:62033441|62044255	0.029774304	0.232918694	#NAME?	down
15:63532647|63563008	0.005214643	0.232918694	#NAME?	down
15:63686359|63689699	0.025004857	0.232918694	Inf	up
15:63693964|63694894	0.046064485	0.232918694	Inf	up
15:63774694|63775649	0.034823636	0.232918694	Inf	up
15:64112573|64123546	0.030955531	0.232918694	Inf	up
15:65729072|65756472	0.001880808	0.232918694	#NAME?	down
15:66535933|66546705	0.013138616	0.232918694	Inf	up
15:67231814|67236820	0.040573467	0.232918694	Inf	up
15:72555271|72567177	0.04640373	0.232918694	Inf	up
15:75407055|75414311	0.020211244	0.232918694	Inf	up
15:75854386|75873568	0.002200649	0.232918694	Inf	up
15:75859878|75883424	0.005484027	0.232918694	Inf	up
15:76292431|76295737	0.021321373	0.232918694	Inf	up
15:78289619|78290575	0.03864058	0.232918694	Inf	up
15:78470532|78471924	0.027017635	0.232918694	Inf	up
15:78544869|78545764	0.033063359	0.232918694	Inf	up
15:78891484|78892313	0.016167174	0.232918694	Inf	up
15:80098416|80122800	0.02252585	0.232918694	#NAME?	down
15:85113873|85117839	0.021523938	0.232918694	Inf	up
15:85684741|85693451	0.000414212	0.232918694	Inf	up
15:90638446|90641199	0.017489723	0.232918694	#NAME?	down
15:92885515|92892072	0.03253428	0.232918694	Inf	up
15:93000512|93004751	0.020888009	0.232918694	#NAME?	down
15:93000512|93014909	0.004328487	0.232918694	Inf	up
16:1724557|1729578	0.006898906	0.232918694	Inf	up
16:1822300|1823037	0.024963874	0.232918694	#NAME?	down
16:3979116|3983440	0.049208872	0.232918694	Inf	up
16:11841695|11842000	0.038134254	0.232918694	Inf	up
16:11894954|11896785	0.007785123	0.232918694	#NAME?	down
16:14627106|14627336	0.031802827	0.232918694	4.69081348	up
16:16007816|16036603	0.035719935	0.232918694	Inf	up
16:24750726|24758599	0.049461038	0.232918694	#NAME?	down
16:27346467|27355907	0.047107923	0.232918694	Inf	up
16:27476451|27486154	0.046908089	0.232918694	Inf	up
16:30483827|30484263	0.019005356	0.232918694	-0.9131805	down
16:30800205|30802470	0.031985411	0.232918694	Inf	up
16:47497399|47547548	0.012429504	0.232918694	#NAME?	down
16:49730771|49731461	0.040652727	0.232918694	Inf	up
16:53267294|53268126	0.013995694	0.232918694	#NAME?	down
16:53873786|53888951	0.009938861	0.232918694	#NAME?	down
16:58599236|58599511	0.008510147	0.232918694	Inf	up
16:68249290|68252653	0.03370853	0.232918694	#NAME?	down
16:70258980|70262524	0.002490704	0.232918694	#NAME?	down
16:71738942|71739341	0.026474535	0.232918694	#NAME?	down

16:72088987|72090786	0.017957373	0.232918694	#NAME?	down
16:84979189|84981994	0.04966905	0.232918694	Inf	up
16:87748673|87762040	0.028680302	0.232918694	#NAME?	down
16:88033109|88038011	7.06E-06	0.048743511	#NAME?	down
17:1361092|1362008	0.02998681	0.232918694	Inf	up
17:2394043|2395454	0.018038472	0.232918694	Inf	up
17:5308694|5332152	0.037007656	0.232918694	Inf	up
17:5308694|5346925	0.020323002	0.232918694	#NAME?	down
17:5354359|5365238	0.010430954	0.232918694	Inf	up
17:8893586|8911507	0.018294916	0.232918694	#NAME?	down
17:21006896|21011309	0.028452767	0.232918694	Inf	up
17:29502589|29510992	0.047989661	0.232918694	Inf	up
17:29648144|29672129	0.014198516	0.232918694	#NAME?	down
17:30481143|30492753	0.049795379	0.232918694	Inf	up
17:31223444|31227606	0.038756852	0.232918694	#NAME?	down
17:31885980|31887313	0.049194305	0.232918694	Inf	up
17:37576341|37585430	0.027720044	0.232918694	-3.6011097	down
17:37584648|37586549	0.003116762	0.232918694	#NAME?	down
17:39423327|39424738	0.042700321	0.232918694	Inf	up
17:40395449|40399131	0.011275889	0.232918694	Inf	up
17:44083545|44083854	0.040869243	0.232918694	Inf	up
17:46170855|46172232	0.043984515	0.232918694	Inf	up
17:47154672|47158303	0.004779773	0.232918694	Inf	up
17:47328269|47345098	0.049769257	0.232918694	Inf	up
17:47370437|47377903	0.018159624	0.232918694	#NAME?	down
17:47742610|47743192	0.016544235	0.232918694	#NAME?	down
17:50139263|50141373	0.046346677	0.232918694	Inf	up
17:56901419|56904488	0.006058611	0.232918694	#NAME?	down
17:58216088|58216736	0.004396659	0.232918694	Inf	up
17:59731421|59735473	0.003722363	0.232918694	#NAME?	down
17:59731421|59773885	0.00035924	0.232918694	#NAME?	down
17:61984171|61985090	0.004251557	0.232918694	Inf	up
17:63792412|63809659	0.046317924	0.232918694	Inf	up
17:64171100|64188415	0.021173877	0.232918694	#NAME?	down
17:65689129|65750724	0.019427942	0.232918694	#NAME?	down
17:67891844|67894165	0.001110209	0.232918694	#NAME?	down
17:68114422|68115178	0.015841349	0.232918694	Inf	up
17:69255543|69256283	0.011325172	0.232918694	Inf	up
17:75937566|75938232	0.040118213	0.232918694	Inf	up
17:80289659|80290728	0.037357723	0.232918694	Inf	up
17:80306252|80306468	0.048977301	0.232918694	Inf	up
17:81270525|81271024	0.000902306	0.232918694	Inf	up
18:2732265|2762236	0.026346988	0.232918694	Inf	up
18:8076455|8114827	0.010851579	0.232918694	Inf	up
18:13037237|13040956	0.026233921	0.232918694	Inf	up
18:21039472|21044186	0.00538363	0.232918694	Inf	up
18:21765772|21804014	0.036747623	0.232918694	#NAME?	down
18:31638644|31666372	0.020969114	0.232918694	Inf	up
18:32111754|32126800	0.04890186	0.232918694	Inf	up
18:42015476|42029441	0.039647046	0.232918694	#NAME?	down
18:42027443|42049605	7.56E-05	0.116074475	Inf	up

18:49331864|49379758	0.041040855	0.232918694	Inf	up
18:50918110|50921187	0.040924039	0.232918694	Inf	up
18:51058125|51059916	0.021707659	0.232918694	Inf	up
18:57613602|57615975	0.04403719	0.232918694	Inf	up
18:69895701|69947532	0.040346849	0.232918694	Inf	up
18:76849526|76868863	0.049389504	0.232918694	Inf	up
18:79096476|79126375	0.024242827	0.232918694	#NAME?	down
19:3623688|3624162	0.02989132	0.232918694	Inf	up
19:4418020|4423874	0.043602241	0.232918694	Inf	up
19:5641594|5641950	0.030659751	0.232918694	Inf	up
19:7938027|7941197	0.047100001	0.232918694	Inf	up
19:10163326|10175618	0.007927025	0.232918694	Inf	up
19:10168330|10177367	0.009373007	0.232918694	Inf	up
19:11724194|11725327	0.044695281	0.232918694	#NAME?	down
19:16088027|16089120	0.005997326	0.232918694	Inf	up
19:18175040|18175697	0.018759241	0.232918694	#NAME?	down
19:18539371|18539720	0.014650635	0.232918694	Inf	up
19:19465340|19474118	0.034471509	0.232918694	-4.072132	down
19:19465345|19474167	0.00932889	0.232918694	Inf	up
19:23662114|23663158	0.015281566	0.232918694	Inf	up
19:24105940|24106643	0.038051159	0.232918694	Inf	up
19:34215124|34215661	0.03670253	0.232918694	Inf	up
19:34221507|34221738	0.042925703	0.232918694	#NAME?	down
19:38343580|38344368	0.014212391	0.232918694	Inf	up
19:38585938|38586191	0.011729121	0.232918694	Inf	up
19:39392464|39393637	0.047359781	0.232918694	Inf	up
19:45263113|45266281	0.041033889	0.232918694	Inf	up
19:48149763|48151339	0.000139389	0.160448253	#NAME?	down
19:50495303|50496608	0.005225792	0.232918694	Inf	up
19:54455364|54455567	0.04945269	0.232918694	#NAME?	down
19:54909245|54910065	0.018739076	0.232918694	Inf	up
19:57455653|57467085	0.048523538	0.232918694	Inf	up
2:3559448|3559729	0.048600751	0.232918694	Inf	up
2:8888018|8958642	0.007987953	0.232918694	Inf	up
2:8908621|8958642	6.71E-09	9.27E-05	6.1046352	up
2:9517901|9543285	0.04067125	0.232918694	Inf	up
2:10644320|10668723	0.044453388	0.232918694	#NAME?	down
2:11455342|11457233	0.021991931	0.232918694	Inf	up
2:24026799|24033257	0.021096154	0.232918694	#NAME?	down
2:24246129|24254431	0.048643413	0.232918694	Inf	up
2:25767583|25771540	0.019430114	0.232918694	#NAME?	down
2:32087492|32089605	0.007851738	0.232918694	Inf	up
2:32126948|32137188	0.048676602	0.232918694	Inf	up
2:32478635|32482582	0.026636932	0.232918694	Inf	up
2:36396614|36442735	0.000545087	0.232918694	#NAME?	down
2:37199704|37201726	0.047756769	0.232918694	Inf	up
2:39056702|39058804	0.026880724	0.232918694	#NAME?	down
2:43912432|43918398	0.03417912	0.232918694	Inf	up
2:44490786|44505730	0.003061792	0.232918694	Inf	up
2:45413114|45419894	0.049567225	0.232918694	Inf	up
2:45546317|45562756	0.045203523	0.232918694	#NAME?	down

2:46974973|46978907	0.041222269	0.232918694	Inf	up
2:47834579|47834871	0.021365013	0.232918694	Inf	up
2:55332566|55335164	0.002476556	0.232918694	Inf	up
2:55643158|55644720	0.023051196	0.232918694	Inf	up
2:61108317|61118116	0.007420803	0.232918694	#NAME?	down
2:63313248|63492940	0.014481827	0.232918694	#NAME?	down
2:70224554|70236175	0.006793105	0.232918694	Inf	up
2:74167014|74172752	0.007859931	0.232918694	Inf	up
2:86121479|86125515	0.042108018	0.232918694	#NAME?	down
2:86147702|86151520	0.049733902	0.232918694	Inf	up
2:88801099|88804880	0.007549015	0.232918694	Inf	up
2:95100474|95101723	0.042840888	0.232918694	#NAME?	down
2:96194958|96195549	0.026308708	0.232918694	Inf	up
2:100006632|100008932	0.010588534	0.232918694	#NAME?	down
2:101842609|101844337	0.043102264	0.232918694	Inf	up
2:105296356|105298705	0.021471527	0.232918694	#NAME?	down
2:108451028|108452466	9.42E-05	0.130086575	Inf	up
2:109564366|109565859	0.040473386	0.232918694	Inf	up
2:111944960|111947567	0.049016219	0.232918694	#NAME?	down
2:120285874|120289757	0.005087185	0.232918694	Inf	up
2:135661270|135680324	0.009203606	0.232918694	Inf	up
2:135748267|135761911	0.031715408	0.232918694	Inf	up
2:143435601|143556485	0.044162723	0.232918694	Inf	up
2:152575136|152581049	0.025145002	0.232918694	#NAME?	down
2:159745770|159748903	5.31E-05	0.104718603	#NAME?	down
2:171011373|171014948	0.045202392	0.232918694	#NAME?	down
2:171014851|171028405	0.02931152	0.232918694	Inf	up
2:177231889|177234271	0.020056137	0.232918694	Inf	up
2:181481598|181485992	0.007217566	0.232918694	Inf	up
2:182952405|182953331	0.003451114	0.232918694	#NAME?	down
2:183128287|183130545	0.016856089	0.232918694	Inf	up
2:196139620|196146055	0.040885246	0.232918694	Inf	up
2:196154968|196163427	0.047653995	0.232918694	Inf	up
2:200856682|200856985	0.040290823	0.232918694	2.88220461	up
2:205158722|205193320	0.049905783	0.232918694	Inf	up
2:208345111|208348023	3.96E-05	0.091475138	Inf	up
2:214767482|214792445	0.035728921	0.232918694	#NAME?	down
2:226864604|226908878	0.002227407	0.232918694	Inf	up
2:229858772|229880128	0.006092704	0.232918694	Inf	up
2:230440476|230450255	0.042157245	0.232918694	#NAME?	down
2:238173534|238185287	0.01658696	0.232918694	Inf	up
2:239126456|239156773	0.004675423	0.232918694	#NAME?	down
2:241654546|241668685	0.044389876	0.232918694	Inf	up
20:9096287|9217452	0.030065206	0.232918694	Inf	up
20:9337126|9339037	0.0244537	0.232918694	Inf	up
20:18297985|18306393	0.039502683	0.232918694	#NAME?	down
20:21326490|21330539	0.039238828	0.232918694	Inf	up
20:31527484|31528050	0.002445958	0.232918694	#NAME?	down
20:34470048|34481206	0.034090774	0.232918694	#NAME?	down
20:35858302|35871829	0.031800167	0.232918694	#NAME?	down
20:35863013|35871829	0.026725039	0.232918694	#NAME?	down

20:37061103|37062270	0.016964652	0.232918694	#NAME?	down
20:44972078|44987296	0.047274478	0.232918694	Inf	up
20:45000392|45001353	0.001658267	0.232918694	#NAME?	down
20:47262288|47276795	0.00265097	0.232918694	#NAME?	down
20:48688657|48691096	0.049340933	0.232918694	Inf	up
20:48690947|48692790	0.001010351	0.232918694	Inf	up
20:49074785|49077064	0.045499963	0.232918694	#NAME?	down
20:51516784|51523596	0.036832614	0.232918694	#NAME?	down
20:62854016|62857635	0.036455304	0.232918694	Inf	up
21:43686804|43688240	0.024655861	0.232918694	Inf	up
21:43998254|43998544	0.011523859	0.232918694	#NAME?	down
22:18126354|18127034	0.005968682	0.232918694	Inf	up
22:21799012|21807846	0.02450622	0.232918694	#NAME?	down
22:28844127|28846512	0.044307705	0.232918694	#NAME?	down
22:30932889|30958694	0.045427563	0.232918694	Inf	up
22:37623327|37623633	0.015744389	0.232918694	#NAME?	down
22:40156115|40251200	0.020350661	0.232918694	Inf	up
22:40273425|40285770	0.045342139	0.232918694	Inf	up
22:40840626|40850880	0.034391128	0.232918694	#NAME?	down
22:41338313|41339191	0.001255079	0.232918694	Inf	up
22:41808875|41810291	0.00697824	0.232918694	Inf	up
22:45178238|45178900	0.028746525	0.232918694	#NAME?	down
22:46997634|47037198	0.018123719	0.232918694	Inf	up
3:4449266|4453049	0.031438706	0.232918694	#NAME?	down
3:15685226|15686309	0.033443183	0.232918694	Inf	up
3:27437388|27448797	0.000424379	0.232918694	Inf	up
3:32733423|32737490	0.041820152	0.232918694	Inf	up
3:33412722|33425741	0.049390042	0.232918694	Inf	up
3:37063742|37083806	0.044103852	0.232918694	Inf	up
3:37083636|37096660	0.04628631	0.232918694	Inf	up
3:37121492|37129149	0.032350631	0.232918694	#NAME?	down
3:40461407|40461661	0.021281462	0.232918694	#NAME?	down
3:47610066|47678311	0.006080615	0.232918694	Inf	up
3:47686049|47693300	0.011252214	0.232918694	Inf	up
3:47720666|47736126	0.014241597	0.232918694	Inf	up
3:49324702|49325845	0.003852601	0.232918694	#NAME?	down
3:52943353|52954411	0.023557803	0.232918694	Inf	up
3:56627037|56628614	0.003228757	0.232918694	6.36350719	up
3:56668397|56673725	0.028758449	0.232918694	Inf	up
3:67495798|67498295	0.04789577	0.232918694	Inf	up
3:71690010|71699708	0.0483455	0.232918694	-0.8857506	down
3:71693875|71699708	0.026135368	0.232918694	Inf	up
3:114500352|114565436	0.040201764	0.232918694	Inf	up
3:119500695|119517315	3.97E-05	0.091475138	#NAME?	down
3:122362968|122365466	0.021726892	0.232918694	Inf	up
3:128262128|128264781	0.002236938	0.232918694	Inf	up
3:132474932|132478140	0.044310168	0.232918694	Inf	up
3:138621407|138622416	0.036581116	0.232918694	#NAME?	down
3:141509568|141540609	0.035625425	0.232918694	Inf	up
3:141512163|141540609	0.044063426	0.232918694	Inf	up
3:149846011|149872154	0.047670112	0.232918694	Inf	up

3:149895473|149921227	0.02455001	0.232918694	Inf	up
3:153164766|153247531	0.04558676	0.232918694	#NAME?	down
3:160411920|160414517	0.038740999	0.232918694	Inf	up
3:168036836|168041474	0.028091164	0.232918694	Inf	up
3:170113360|170136665	0.030033352	0.232918694	#NAME?	down
3:170128684|170149244	0.03948997	0.232918694	#NAME?	down
3:170145423|170178938	0.011057959	0.232918694	#NAME?	down
3:172251260|172298787	0.037229203	0.232918694	Inf	up
3:182961226|182965753	0.025416324	0.232918694	Inf	up
3:183086693|183092545	0.038085079	0.232918694	Inf	up
3:183205876|183207823	0.010733723	0.232918694	Inf	up
3:183643480|183651276	0.008715005	0.232918694	#NAME?	down
3:195291706|195308837	0.04596421	0.232918694	Inf	up
3:195295708|195302174	0.028686119	0.232918694	Inf	up
3:195301575|195302174	0.046561747	0.232918694	Inf	up
3:195333028|195357784	0.040856546	0.232918694	Inf	up
3:195516842|195524896	0.007560178	0.232918694	#NAME?	down
3:196806579|196812851	0.001512985	0.232918694	#NAME?	down
3:197830770|197839397	0.032459349	0.232918694	#NAME?	down
3:197865423|197866219	0.014394856	0.232918694	-5.3392127	down
3:197866112|197871462	0.001108775	0.232918694	#NAME?	down
4:2954424|2956811	0.019577582	0.232918694	#NAME?	down
4:15816511|15825016	0.019899496	0.232918694	Inf	up
4:25154998|25159107	0.013290121	0.232918694	#NAME?	down
4:40144632|40154267	0.046718598	0.232918694	Inf	up
4:40890364|40935139	0.038871763	0.232918694	Inf	up
4:41013583|41014398	0.015947029	0.232918694	#NAME?	down
4:42503450|42507154	0.039507808	0.232918694	#NAME?	down
4:42503450|42524847	0.001706504	0.232918694	Inf	up
4:42503450|42543986	0.022434562	0.232918694	Inf	up
4:48642277|48646017	0.039478166	0.232918694	Inf	up
4:51863437|51899378	0.036535315	0.232918694	#NAME?	down
4:53414615|53444103	0.031633041	0.232918694	Inf	up
4:53425872|53453133	0.025964294	0.232918694	Inf	up
4:56011412|56011985	0.006133266	0.232918694	#NAME?	down
4:67798639|67824295	0.048270207	0.232918694	Inf	up
4:71486948|71497692	0.008749341	0.232918694	#NAME?	down
4:77058507|77066405	0.008710402	0.232918694	#NAME?	down
4:82824555|82827632	0.03280205	0.232918694	#NAME?	down
4:82871944|82875822	0.013851956	0.232918694	Inf	up
4:83446835|83448961	0.039756518	0.232918694	Inf	up
4:88419302|88420428	0.008161586	0.232918694	#NAME?	down
4:99028867|99048876	0.024130837	0.232918694	Inf	up
4:105396249|105424322	0.043498692	0.232918694	#NAME?	down
4:118088039|118114960	0.014760606	0.232918694	#NAME?	down
4:127921524|127939997	0.039693211	0.232918694	Inf	up
4:143221175|143227030	0.049086519	0.232918694	Inf	up
4:151174670|151187463	0.004969084	0.232918694	#NAME?	down
4:152953499|152954319	0.015686515	0.232918694	#NAME?	down
4:153626147|153632838	0.008174671	0.232918694	#NAME?	down
4:155774968|155777642	0.003155969	0.232918694	Inf	up

4:159238809|159243791	0.041488309	0.232918694	Inf	up
5:6604138|6605408	0.016067729	0.232918694	#NAME?	down
5:34818797|34821850	0.049342304	0.232918694	Inf	up
5:37114960|37120340	0.038943026	0.232918694	Inf	up
5:37213559|37224740	0.038871763	0.232918694	Inf	up
5:43675511|43677806	0.024359699	0.232918694	2.68028285	up
5:43675511|43704400	0.011075458	0.232918694	#NAME?	down
5:50750151|50763242	0.042252644	0.232918694	#NAME?	down
5:57230846|57231321	0.001757649	0.232918694	#NAME?	down
5:61472681|61473037	0.004941386	0.232918694	#NAME?	down
5:65224320|65226219	0.037107905	0.232918694	#NAME?	down
5:65508067|65554946	0.043676314	0.232918694	Inf	up
5:65567513|65572286	0.010689385	0.232918694	Inf	up
5:69306817|69314043	0.021664871	0.232918694	#NAME?	down
5:71502599|71512428	0.043502243	0.232918694	Inf	up
5:71548682|71549606	0.04478478	0.232918694	Inf	up
5:75384642|75386034	0.043512488	0.232918694	Inf	up
5:77046347|77048272	0.009934873	0.232918694	-4.3120815	down
5:88804598|88823927	0.049042101	0.232918694	#NAME?	down
5:90495677|90506674	0.005864078	0.232918694	Inf	up
5:94628811|94629167	0.044262041	0.232918694	Inf	up
5:94643273|94654752	0.003028309	0.232918694	Inf	up
5:97098937|97103094	0.00903939	0.232918694	Inf	up
5:98879552|98881375	0.040914963	0.232918694	Inf	up
5:113001084|113001674	0.043888258	0.232918694	Inf	up
5:119240419|119244576	0.010806356	0.232918694	Inf	up
5:122826126|122827646	0.040720642	0.232918694	Inf	up
5:129104956|129107062	0.002135855	0.232918694	Inf	up
5:131180095|131187109	0.042921167	0.232918694	Inf	up
5:133006759|133006920	0.039621828	0.232918694	#NAME?	down
5:134666823|134671886	0.045142174	0.232918694	Inf	up
5:137634037|137639977	0.007126703	0.232918694	Inf	up
5:137692285|137698408	0.047503953	0.232918694	Inf	up
5:137952628|137954376	0.018037032	0.232918694	#NAME?	down
5:139317053|139319501	0.047749626	0.232918694	Inf	up
5:140440119|140445975	0.018023912	0.232918694	Inf	up
5:146254943|146258593	0.005682604	0.232918694	#NAME?	down
5:169681744|169684350	0.021097613	0.232918694	Inf	up
5:172057349|172061268	0.048938825	0.232918694	Inf	up
5:177244195|177246796	0.030947975	0.232918694	Inf	up
5:177536358|177539147	0.021304051	0.232918694	-4.75922	down
5:179580947|179589647	0.020740586	0.232918694	Inf	up
5:179591625|179593645	0.033050146	0.232918694	Inf	up
6:2248837|2263684	0.028855688	0.232918694	#NAME?	down
6:3085259|3104315	0.002676161	0.232918694	Inf	up
6:6248312|6250929	0.012774371	0.232918694	Inf	up
6:7176655|7182082	0.033275123	0.232918694	Inf	up
6:7176655|7189322	0.039045193	0.232918694	-3.142908	down
6:11192345|11193692	0.049454243	0.232918694	Inf	up
6:13652659|13696896	0.033758184	0.232918694	Inf	up
6:15410224|15468718	0.038539985	0.232918694	Inf	up

6:18212488|18213781	0.031844118	0.232918694	Inf	up
6:18256361|18258405	0.025093334	0.232918694	Inf	up
6:31269966|31354665	0.040891921	0.232918694	#NAME?	down
6:32520164|32580856	0.040482027	0.232918694	Inf	up
6:33085779|33128098	0.001408465	0.232918694	Inf	up
6:36158648|36158941	0.033144743	0.232918694	Inf	up
6:36524341|36540207	0.000402196	0.232918694	Inf	up
6:42592151|42606651	0.04596421	0.232918694	Inf	up
6:42676056|42679832	0.008014481	0.232918694	Inf	up
6:46881455|46883665	0.031033774	0.232918694	Inf	up
6:47526628|47554766	0.035793546	0.232918694	#NAME?	down
6:73473365|73480126	0.045397805	0.232918694	Inf	up
6:75634707|75678927	0.000755908	0.232918694	#NAME?	down
6:78965703|78985428	0.016209242	0.232918694	Inf	up
6:85618818|85624112	0.028524828	0.232918694	Inf	up
6:90568558|90571807	0.000918785	0.232918694	Inf	up
6:104843223|104849246	0.01556177	0.232918694	Inf	up
6:107904629|107924932	0.045196226	0.232918694	Inf	up
6:110626776|110627145	0.041840161	0.232918694	Inf	up
6:111262257|111263946	0.042645144	0.232918694	Inf	up
6:111405470|111472140	0.048808325	0.232918694	Inf	up
6:116698704|116705160	0.007228601	0.232918694	4.61724891	up
6:118479871|118483858	0.038336286	0.232918694	Inf	up
6:127812883|127813931	0.041269115	0.232918694	#NAME?	down
6:129611533|129618852	0.042641521	0.232918694	Inf	up
6:130022227|130120958	0.017009385	0.232918694	#NAME?	down
6:131164398|131169273	0.048878006	0.232918694	Inf	up
6:135039573|135042126	0.004057877	0.232918694	#NAME?	down
6:136388393|136389517	0.023140319	0.232918694	Inf	up
6:136669283|136698682	0.021072355	0.232918694	Inf	up
6:136694140|136698682	0.007001154	0.232918694	Inf	up
6:138907495|138908816	0.010722741	0.232918694	#NAME?	down
6:144490071|144493456	0.049841405	0.232918694	Inf	up
6:149765691|149773169	0.027931882	0.232918694	Inf	up
6:150795856|150800869	0.045370229	0.232918694	Inf	up
6:154773989|154795139	0.000569298	0.232918694	#NAME?	down
6:154860738|154860998	0.021255813	0.232918694	Inf	up
6:158282263|158314268	0.000189527	0.192998461	#NAME?	down
6:167022409|167033971	0.024493204	0.232918694	Inf	up
7:579256|607452	0.008338892	0.232918694	Inf	up
7:4983671|4989177	0.015317277	0.232918694	Inf	up
7:6585016|6585260	0.015353226	0.232918694	Inf	up
7:8022483|8071131	0.049676415	0.232918694	Inf	up
7:16216066|16278226	0.041825733	0.232918694	Inf	up
7:17845595|17850964	0.027194004	0.232918694	3.6465317	up
7:23123777|23144025	0.041250455	0.232918694	#NAME?	down
7:33357855|33388144	0.017579185	0.232918694	Inf	up
7:35879823|35885879	0.019112251	0.232918694	#NAME?	down
7:38242428|38260230	0.043229704	0.232918694	#NAME?	down
7:39997494|40002031	0.035306794	0.232918694	#NAME?	down
7:39997494|40047877	0.02325452	0.232918694	Inf	up

7:50319048|50382707	0.047367655	0.232918694	Inf	up
7:64336259|64336982	0.033953834	0.232918694	Inf	up
7:64937342|64937630	0.003359487	0.232918694	Inf	up
7:65979399|65980409	0.01693856	0.232918694	Inf	up
7:66127704|66134374	0.020793818	0.232918694	-3.6034095	down
7:69899286|69899498	0.038233477	0.232918694	Inf	up
7:72831602|72831798	0.030625562	0.232918694	Inf	up
7:73459536|73470483	0.04923845	0.232918694	Inf	up
7:77571078|77597901	0.00130919	0.232918694	Inf	up
7:77585543|77600806	0.014582921	0.232918694	2.1579576	up
7:77585543|77607301	0.020329981	0.232918694	Inf	up
7:90726567|90726812	0.033739155	0.232918694	Inf	up
7:90726567|90747775	0.023059017	0.232918694	Inf	up
7:92079079|92080152	0.007941076	0.232918694	Inf	up
7:92221902|92222978	0.040420152	0.232918694	Inf	up
7:92294889|92307656	0.045927331	0.232918694	-2.5541772	down
7:92307359|92327900	0.042054108	0.232918694	Inf	up
7:92457552|92492286	0.013294743	0.232918694	#NAME?	down
7:98190728|98194572	0.016409516	0.232918694	#NAME?	down
7:100328836|100332576	0.032701622	0.232918694	Inf	up
7:103128483|103128792	0.025542042	0.232918694	Inf	up
7:103353921|103355793	0.040406042	0.232918694	#NAME?	down
7:105090009|105091314	0.013816879	0.232918694	Inf	up
7:105665066|105665288	0.022418911	0.232918694	Inf	up
7:106872537|106886292	0.043117842	0.232918694	Inf	up
7:112286873|112287074	6.81E-05	0.116074475	Inf	up
7:121170582|121266779	0.006046132	0.232918694	#NAME?	down
7:128642217|128648982	0.030763664	0.232918694	Inf	up
7:130039464|130049144	0.045981009	0.232918694	#NAME?	down
7:130170400|130181719	0.04158064	0.232918694	#NAME?	down
7:131387120|131388972	0.035716961	0.232918694	Inf	up
7:138519189|138551180	0.00507146	0.232918694	Inf	up
7:139266333|139272440	0.015100049	0.232918694	#NAME?	down
7:139409563|139412580	0.020481812	0.232918694	#NAME?	down
7:155672867|155685052	0.00051676	0.232918694	#NAME?	down
7:155700773|155707068	0.044202455	0.232918694	#NAME?	down
7:155711173|155718443	0.025849746	0.232918694	Inf	up
7:156826605|156836885	0.002514351	0.232918694	Inf	up
7:157201721|157231327	0.033015634	0.232918694	Inf	up
7:158876609|158891333	0.002759737	0.232918694	Inf	up
8:690898|692612	0.008151094	0.232918694	#NAME?	down
8:8154187|8154530	0.045455849	0.232918694	Inf	up
8:25407983|25408940	0.029639034	0.232918694	#NAME?	down
8:30101898|30104486	0.00244176	0.232918694	Inf	up
8:37870420|37877551	0.014567011	0.232918694	Inf	up
8:38110379|38110833	0.023432537	0.232918694	Inf	up
8:38114192|38116725	0.00261005	0.232918694	Inf	up
8:38114192|38121149	0.016106187	0.232918694	#NAME?	down
8:38133454|38135766	0.028202043	0.232918694	#NAME?	down
8:39021643|39023325	0.034326194	0.232918694	Inf	up
8:42957094|42964474	0.039446555	0.232918694	Inf	up

8:43002107|43013400	0.033797151	0.232918694	Inf	up
8:51857460|51861246	0.043854379	0.232918694	Inf	up
8:61633683|61653660	0.028858048	0.232918694	Inf	up
8:67093543|67095732	0.001345897	0.232918694	-4.5560662	down
8:67103037|67137603	0.032510834	0.232918694	Inf	up
8:68022036|68038292	0.026718665	0.232918694	#NAME?	down
8:73688654|73738349	0.047366152	0.232918694	Inf	up
8:100287501|100288267	0.035648116	0.232918694	Inf	up
8:109409865|109420690	0.035101132	0.232918694	Inf	up
8:123140761|123144855	0.035676369	0.232918694	Inf	up
8:123337625|123339446	0.036612225	0.232918694	Inf	up
8:129903312|129904585	0.020514265	0.232918694	Inf	up
8:130236922|130358143	0.034076667	0.232918694	#NAME?	down
8:140739018|140752316	0.008005437	0.232918694	Inf	up
8:140818277|140864399	0.02163994	0.232918694	Inf	up
8:140830472|140890769	0.046962413	0.232918694	#NAME?	down
8:144791773|144792140	0.046715814	0.232918694	Inf	up
9:271627|289581	0.022531699	0.232918694	Inf	up
9:2161686|2161903	0.046244654	0.232918694	#NAME?	down
9:6477622|6482099	0.045728528	0.232918694	Inf	up
9:14639896|14680162	0.047695529	0.232918694	#NAME?	down
9:17330632|17342444	0.000687387	0.232918694	#NAME?	down
9:20413721|20414425	0.027265823	0.232918694	Inf	up
9:26984257|27011968	0.044200345	0.232918694	Inf	up
9:33953285|33963791	0.006708146	0.232918694	Inf	up
9:33960826|33989126	0.033374523	0.232918694	1.75082591	up
9:33986760|33989126	0.016623591	0.232918694	#NAME?	down
9:34269257|34271930	0.046918408	0.232918694	Inf	up
9:70053992|70054173	0.048858487	0.232918694	Inf	up
9:71690092|71698204	0.031434337	0.232918694	Inf	up
9:77922161|77935889	0.047857004	0.232918694	Inf	up
9:79573689|79654075	0.048656973	0.232918694	Inf	up
9:85974967|85978381	0.037488899	0.232918694	#NAME?	down
9:86033282|86046572	0.045575208	0.232918694	Inf	up
9:94923986|94956015	0.008234826	0.232918694	Inf	up
9:96320998|96324169	0.027268444	0.232918694	Inf	up
9:96465337|96465778	0.019479184	0.232918694	#NAME?	down
9:104722868|104723718	0.032259266	0.232918694	Inf	up
9:109050283|109064567	0.025840605	0.232918694	Inf	up
9:121171159|121171548	0.04603178	0.232918694	Inf	up
9:122280443|122291840	0.001586874	0.232918694	Inf	up
9:123169902|123173842	0.001853127	0.232918694	#NAME?	down
9:123179007|123184298	0.007817991	0.232918694	#NAME?	down
9:123757703|123879021	0.020949362	0.232918694	#NAME?	down
9:124899329|124900547	0.039636652	0.232918694	#NAME?	down
9:128718325|128718625	0.042613225	0.232918694	Inf	up
9:128907157|128909321	0.008972958	0.232918694	#NAME?	down
9:129098013|129098650	0.031926991	0.232918694	Inf	up
9:135947798|135953753	0.048306705	0.232918694	Inf	up
9:137790848|137800984	0.033635658	0.232918694	Inf	up
X:2719661|2726373	0.008885733	0.232918694	#NAME?	down

X:17103718|17138961	0.017984135	0.232918694	Inf	up
X:19050806|19050981	0.022442845	0.232918694	Inf	up
X:24172715|24179770	0.029223043	0.232918694	#NAME?	down
X:37386599|37426000	0.035302846	0.232918694	Inf	up
X:51327721|51357027	0.043420974	0.232918694	Inf	up
X:53645311|53654131	0.029077251	0.232918694	Inf	up
X:65075606|65113813	0.038439585	0.232918694	#NAME?	down
X:67116173|67116338	0.045634816	0.232918694	Inf	up
X:68192919|68201710	0.005947	0.232918694	Inf	up
X:78014662|78021079	0.023434484	0.232918694	#NAME?	down
X:118442244|118443687	0.04836177	0.232918694	Inf	up
X:118542725|118543593	0.010078355	0.232918694	Inf	up
X:119629318|119629508	2.74E-05	0.091475138	Inf	up
X:121090399|121093477	0.002260396	0.232918694	#NAME?	down
X:132281095|132283032	0.046309991	0.232918694	Inf	up
X:148651999|148662768	0.0137521	0.232918694	Inf	up
X:155415575|155419562	0.006508547	0.232918694	Inf	up
X:155506898|155537118	0.034817087	0.232918694	Inf	up
Y:2961074|2961646	0.04528401	0.232918694	Inf	up
Y:12709388|12722187	0.002719312	0.232918694	#NAME?	down
